# Hierarchical interface engineering for advanced magnesium-based hydrogen storage: synergistic effects of structural design and compositional modification

**DOI:** 10.1039/d5sc01169h

**Published:** 2025-04-01

**Authors:** Han Jiang, Zhao Ding, Yuting Li, Guo Lin, Shaoyuan Li, Wenjia Du, Yu'an Chen, Leon L. Shaw, Fusheng Pan

**Affiliations:** a College of Materials Science and Engineering, National Engineering Research Center for Magnesium Alloys, National Innovation Centre for Industry-Education Integration of Energy Storage Technology, Chongqing University Chongqing China zhaoding@cqu.edu.cn; b Chongqing Institute of New Energy Storage Materials and Equipment Chongqing China; c State Key Laboratory of Complex Nonferrous Metal Resources Clean Utilization, Faculty of Metallurgical and Energy Engineering, Kunming University of Science and Technology Kunming China; d Department of Engineering Science, University of Oxford Oxford UK; e Department of Mechanical, Materials and Aerospace Engineering, Illinois Institute of Technology Chicago USA

## Abstract

Interface engineering fundamentally revolutionizes magnesium-based hydrogen storage systems by orchestrating atomic-scale interactions and mass transport pathways through precisely engineered structural architectures and chemical environments. This review presents a paradigm-shifting framework that transcends conventional surface modification approaches, establishing interface engineering as a cornerstone strategy for next-generation hydrogen storage materials. Through sophisticated control of interface architecture – from one-dimensional confined channels that facilitate directional hydrogen diffusion, to two-dimensional platforms that maximize catalytic interactions, to three-dimensional networks that optimize spatial organization – we unlock unprecedented control over hydrogen storage dynamics. The strategic modulation of interface chemistry creates synergistic effects between structural features and catalytic functionalities. Metal–metal interfaces orchestrate electron transfer processes and facilitate hydrogen dissociation, while engineered support interfaces maintain structural integrity and enhance cycle life. This multi-level interface control enables simultaneous optimization of thermodynamic destabilization and kinetic enhancement. Advanced characterization and theoretical modeling reveal that the controlled evolution of interface structure during hydrogen cycling plays a pivotal role in determining long-term performance stability. Our comprehensive analysis establishes fundamental correlations between interface architecture and hydrogen storage mechanisms, providing critical insights for rational material design. The review concludes by identifying key challenges and opportunities in translating these interface engineering principles into practical energy storage technologies, offering a roadmap for future development of high-performance magnesium-based hydrogen storage systems.

## Abstract 2

Interface engineering fundamentally revolutionizes magnesium-based hydrogen storage systems by orchestrating atomic-scale interactions and mass transport pathways through synergistic integration of structural design and compositional modification.

## Graphical abstract

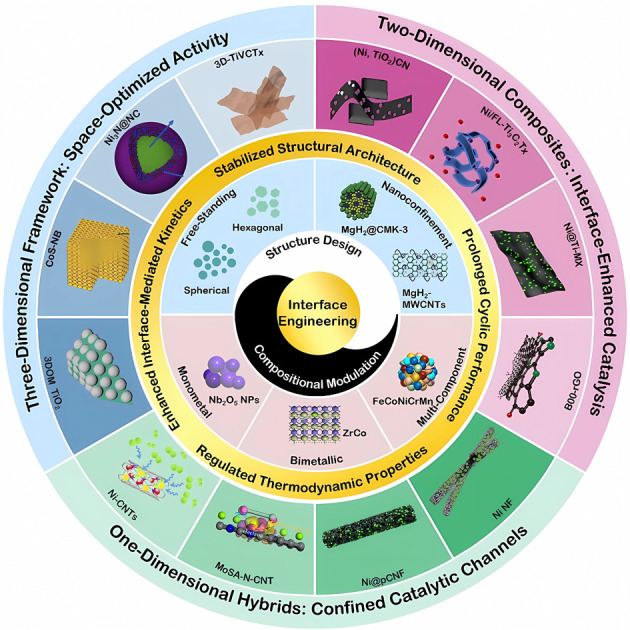

## 1. Introduction

Global energy consumption patterns and environmental challenges have reached an unprecedented turning point, fundamentally reshaping our approach to sustainable development.^1–5^ The trajectory of worldwide energy utilization mirrors the evolution of industrial civilizations – from the First Industrial Revolution's coal-driven steam engines to the Second Industrial Revolution's oil-based internal combustion engines, each technological leap has been accompanied by increasing environmental costs.^6,7^ Current projections indicate that global energy demand will double by 2050, creating an urgent imperative for transformative solutions. This expansion, primarily driven by rapid industrialization in developing nations and increasing energy intensity in developed economies, poses unprecedented challenges to environmental sustainability. Analysis of historical energy consumption trends reveals a critical inflection point: while fossil fuels have powered remarkable technological progress, their continued dominance threatens to push global ecosystems beyond critical thresholds. Under existing utilization patterns, annual greenhouse gas emissions are projected to exceed the carbon budget required for maintaining global temperature rise below 2 °C by 2030.^8,9^ This environmental imperative is further complicated by the projected persistence of traditional energy sources, with oil consumption expected to maintain significant market share through 2035. The convergence of these factors – escalating energy demand, environmental constraints, and the inertia of existing energy systems – necessitates immediate transition toward sustainable energy paradigms. The emergence of hydrogen-based energy systems offers exceptional potential for addressing these challenges through their unique combination of high gravimetric energy density (142 MJ kg^−1^, nearly three times that of gasoline) and zero-emission characteristics.^10–13^

**Fig. 1:**
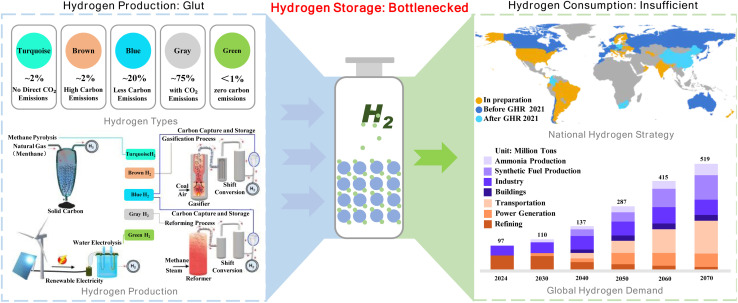
Schematic illustration of the hydrogen industry.^14–19^ GHR: Global Hydrogen Review.

As shown in Fig. 1, the hydrogen economy encompasses three fundamental aspects: hydrogen production, storage, and utilization. The taxonomy of hydrogen production, illustrated in the top-left section, categorizes hydrogen into five types based on carbon emissions and production methodologies, with pathways ranging from methane pyrolysis and coal gasification to renewable-powered water electrolysis.^14^ The global hydrogen strategy landscape, illustrated in the upper-right section of Fig. 1, reveals the evolutionary trajectory of national hydrogen initiatives worldwide. As of September 2021, countries with established national hydrogen strategies (shown in blue), including the European Union and 16 other nations, collectively accounted for approximately 20% of global energy-related CO_2_ emissions.^15^ The bar chart in the lower right illustrates the growth trend of global hydrogen demand, rising from 9700 tons in 2024 to a projected 28 700 tons by 2050. This reflects the rapid increase in future hydrogen demand and vividly showcases the expansion of the hydrogen market.^16^ Meanwhile, major economies such as China (indicated in light blue) and the United States (marked in orange) have subsequently accelerated their strategic planning. Notably, in March 2022, China formalized its commitment by releasing the “Medium and Long-term Plan for the Development of the Hydrogen Industry (2021–2035)” through the National Development and Reform Commission (NDRC), signaling a comprehensive approach to hydrogen energy development. Despite the promising prospects of hydrogen-based energy systems, the development of efficient and reliable hydrogen storage technologies remains a critical bottleneck in advancing the hydrogen economy,^17–19^ which constitute the central bottleneck illustrated in Fig. 1. Conventional storage approaches, particularly high-pressure gas systems (350–700 bar), while commercially established, face significant limitations in terms of volumetric efficiency and safety considerations, requiring sophisticated infrastructure and rigorous safety protocols.^20–24^ Similarly, cryogenic liquid storage (−253 °C) demands substantial energy input for liquefaction and complex thermal management systems, making it economically challenging for widespread implementation.^25,26^ These limitations have driven research toward alternative storage solutions, particularly solid-state materials, which show potential for addressing the key challenges in hydrogen storage technology.^27–29^ The solid-state hydrogen storage approach offers compelling advantages through the integration of superior volumetric hydrogen density under moderate conditions, enhanced operational safety through stable chemical bonds, and improved system integration potential.^30–32^ By storing hydrogen through chemical bonding rather than physical containment, this methodology circumvents many engineering challenges associated with high-pressure and cryogenic systems while offering significant cost advantages through simplified infrastructure requirements.

Among solid-state hydrogen storage materials, magnesium-based systems have emerged as particularly promising candidates due to their exceptional combination of advantages: high theoretical storage capacity (7.6 wt% H_2_), natural abundance and environmental compatibility.^33^ Their remarkable gravimetric density, nearly twice that of conventional metal hydrides, coupled with magnesium's cost-effectiveness and material sustainability, positions these systems as prime candidates for practical large-scale implementation. However, two fundamental scientific challenges have hindered their widespread adoption. First, the high thermodynamic stability (Δ*H* = 74.7 kJ per mol H_2_)^34^ necessitates elevated operating temperatures (>300 °C) for hydrogen release, making thermal management challenging for mobile applications. Second, the inherent kinetic limitations stemming from substantial energy barriers for both hydrogen dissociation at the surface and atomic diffusion through the hydride phase significantly restrict practical sorption rates under moderate conditions.^35^ Recent breakthroughs in nanomaterial synthesis and advanced characterization techniques have revealed that interfacial phenomena – particularly electron transfer at metal–metal interfaces and selective mass transport through engineered channels – play crucial roles in determining hydrogen storage performance.^36,37^ These findings suggest that rational design of material interfaces could provide effective pathways for simultaneously addressing both thermodynamic and kinetic limitations. While significant progress has been achieved in laboratory-scale demonstrations through various interface engineering strategies, translating these advances into practical energy storage systems requires systematic investigation across multiple dimensions.

This review presents a systematic analysis of interface engineering strategies in magnesium-based hydrogen storage materials, examining their evolution from fundamental principles to practical applications. The conceptual framework positions interface engineering as a cornerstone strategy built upon the synergistic integration of structural design and compositional modulation. Our comprehensive approach examines the progression of interface architectures across distinct dimensional domains: from one-dimensional confined channels providing efficient diffusion pathways, to two-dimensional platforms offering extensive catalytic interfaces, and ultimately to three-dimensional frameworks achieving comprehensive performance enhancement through optimized spatial organization. In parallel, we analyze how strategic incorporation of catalytic components and electronic modification creates chemically active interfaces that significantly enhance reaction kinetics. This dual-functional design approach enables four key performance attributes: regulated thermodynamic properties through interface-mediated destabilization, enhanced reaction kinetics *via* optimized catalytic pathways, stabilized structural architecture maintaining long-term cycling capability, and prolonged performance through controlled interface evolution. Throughout the review, we establish fundamental correlations between interface architecture and hydrogen storage mechanisms while identifying critical challenges and opportunities for developing practical magnesium-based hydrogen storage systems through rational interface design.

## 2. Physicochemical fundamentals and limiting mechanisms

The evolution of metal hydride science traces back to Graham's pioneering discovery in 1866,^38^ where the unique metal–hydrogen interaction mechanisms were first revealed, establishing the foundation for modern gas–solid storage systems. In magnesium-based hydrogen storage, performance is governed by the intricate interplay between electronic structure, crystallographic transformations, and interfacial phenomena.^39^ Understanding these fundamental aspects is crucial as they collectively determine the system's practical viability: electronic configurations dictate hydrogen binding energetics, crystal structure evolution controls hydrogen diffusion pathways, while interfacial processes dominate reaction kinetics. This comprehensive analysis of physicochemical principles not only explains the exceptional theoretical capacity (7.6 wt% H_2_) but also reveals the origins of practical limitations – notably the high thermodynamic stability (Δ*H* = 74.7 kJ per mol H_2_) and substantial kinetic barriers for hydrogen dissociation and diffusion. These fundamental characteristics establish the framework for developing effective interface engineering strategies, where precise control of local electronic states and atomic arrangements can potentially overcome inherent performance limitations while maintaining the system's advantageous storage properties.

### 2.1. Crystal structure and electronic features: origin of storage behavior

The hydrogen storage characteristics of the Mg/MgH_2_ system originate from its unique crystallographic architecture and electronic configuration – two fundamental aspects that govern its storage behavior at both atomic and molecular levels. The intricate interplay between structural transformations and electronic state evolution during hydrogenation–dehydrogenation cycles determines key performance metrics including storage capacity, reaction kinetics, and cycling stability. Understanding these structure–property relationships is essential for developing effective interface engineering strategies, as the local atomic arrangements and electronic configurations at interfaces critically influence hydrogen dissociation, diffusion, and recombination processes.

**Fig. 2:**
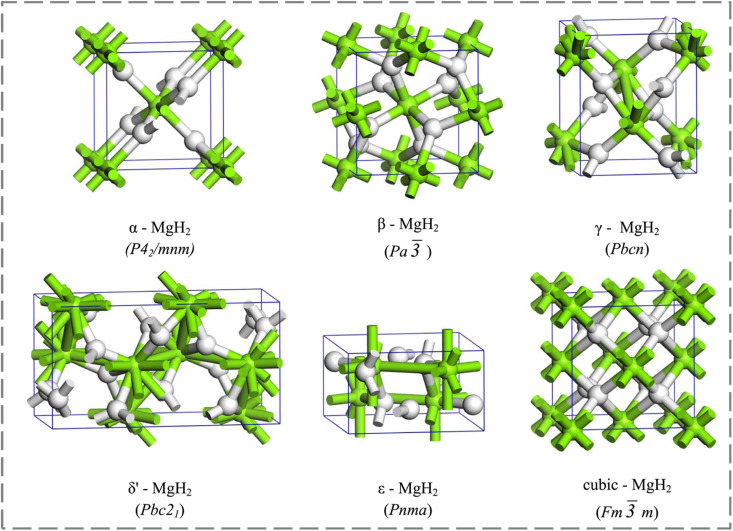
The MgH_2_ structures investigated in the present study.^42^

The structural evolution of the Mg/MgH_2_ system exhibits sophisticated phase transformation behavior governed by thermodynamic conditions and crystallographic constraints. In its initial state, pure magnesium adopts a hexagonal close-packed (HCP) structure,^40^ characterized by highly ordered atomic arrangements at hexagonal prism vertices and face centers that provide potential sites for hydrogen incorporation. Upon hydrogenation, the system undergoes a dramatic structural reconstruction to form tetragonal α-MgH_2_ (space group *P*4_2_/*mnm*), accompanied by substantial volumetric expansion (∼32%) that generates significant internal strain fields critical for subsequent hydrogen storage dynamics. This base structure demonstrates remarkable polymorphic flexibility under varying pressure conditions, revealing a series of phase transitions that fundamentally influence hydrogen storage behavior. At pressures above 3.9 kbar, γ-MgH_2_ emerges with an orthorhombic α-PbO_2_-type structure, followed by transformation to cubic β-MgH_2_ (CaF_2_-type) beyond 38.4 kbar, and ultimately the formation of δ′-MgH_2_ above 102.6 kbar at room temperature.^41^ Detailed crystallographic analysis reveals systematic variations in Mg–H bonding configurations across these polymorphs: α-MgH_2_ features two distinct bond types essential for hydrogen storage, γ-MgH_2_ exhibits three unique configurations that enhance structural flexibility, β-MgH_2_ shows singular bonding characteristics, while δ′-MgH_2_ presents seven different arrangements that significantly impact hydrogen diffusion pathways. Additionally, at elevated pressures above 5 GPa, ε-MgH_2_ (*Pnma*) forms with enhanced structural stability due to its dense orthorhombic configuration, while cubic MgH_2_ (*Fm*3̄*m*) appears beyond 15 GPa, featuring a closely packed hydrogen sublattice that fundamentally alters the system's storage properties (Fig. 2).^42^ The systematic evolution of these crystallographic phases and their associated bonding characteristics provides crucial insights into the structure–property relationships governing hydrogen storage performance, establishing a fundamental framework for interface engineering strategies.

The electronic landscape undergoes profound transformation during the hydrogenation process, exhibiting characteristics that fundamentally dictate hydrogen storage behavior. The system transitions from metallic magnesium's characteristic conductive state to MgH_2_'s semiconducting nature, accompanied by a substantial increase in band gap. This electronic reconstruction is driven by sophisticated orbital interactions between hydrogen s-states and magnesium p-orbitals, resulting in the formation of Mg–H bonds with an optimal length of 1.93 Å.^43^ The bonding mechanism involves significant charge redistribution: magnesium atoms develop partial positive charges while hydrogen sites acquire negative character, establishing a complex ionic-covalent network. This electronic configuration, while enhancing structural stability, simultaneously creates substantial energy barriers for hydrogen release. The transformation in electronic structure manifests particularly in the system's electrical conductivity, dramatically impacting charge transfer processes and hydrogen diffusion pathways, thereby governing the overall kinetic behavior of hydrogen storage processes. Understanding these electronic features is crucial for interface engineering, as they determine the nature of catalytic interactions and charge transfer processes at material interfaces.

The intricate interplay between crystallographic architecture and electronic structure in the Mg/MgH_2_ system reveals several key design principles for interface engineering. The polymorphic nature of MgH_2_ offers multiple pathways for structural optimization through interface control, while the electronic transitions suggest opportunities for modifying energy barriers through strategic introduction of catalytic interfaces. These fundamental characteristics guide the development of advanced interface engineering strategies: structural features inform the design of physical interfaces for stabilizing specific phases, while electronic properties direct the selection of catalytic components for enhancing reaction kinetics. This comprehensive understanding of structure–electronic–property relationships establishes the foundation for rational design of high-performance magnesium-based hydrogen storage materials through sophisticated interface architecture.

### 2.2. Kinetic analysis: rate-controlling steps and barriers

The hydrogen storage behavior of Mg/MgH_2_ system involves complex heterogeneous gas–solid reactions characterized by multiple sequential and parallel processes, each with distinct kinetic barriers and rate-determining mechanisms.^44–47^ While the overall reaction can be represented by the fundamental equation

1 Mg + H_2_ ↔ MgH_2_ + 75 kJ per mol H_2_

The actual storage process encompasses a series of elementary steps including surface adsorption, molecular dissociation, atomic diffusion, and phase transformation.^48^ The high formation enthalpy (75 kJ per mol H_2_) reflects not only the system's inherent thermodynamic stability but also indicates substantial kinetic barriers that necessitate temperatures above 300 °C for efficient hydrogen release. Understanding these multi-step processes and their associated energy barriers is crucial for developing effective interface engineering strategies.

**Fig. 3:**
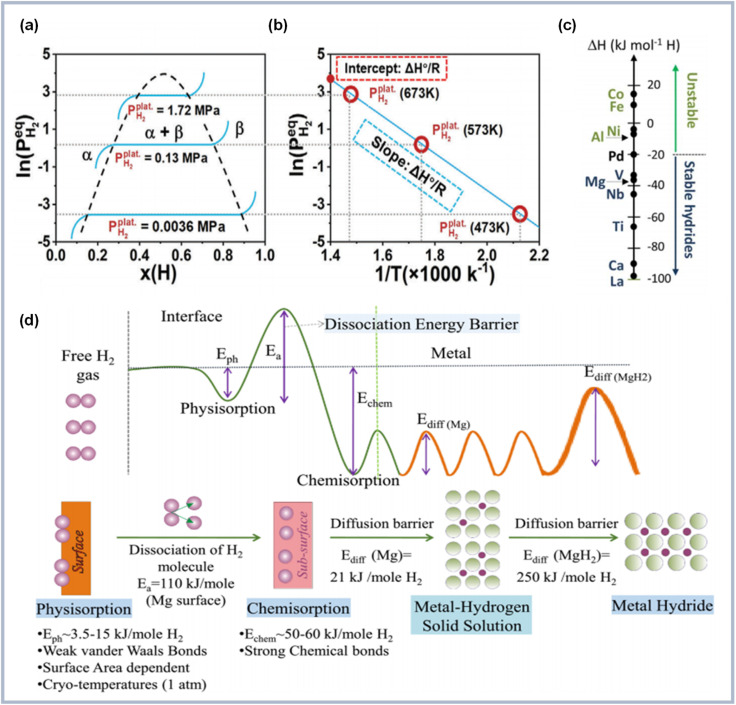
(a) Pressure–composition isotherm plot of Mg + H_2_ ⇄ MgH_2_ transition (b) van't Hoff plot related to the phase transition of Mg + H_2_ ⇄ MgH_2_;^49^ (c) enthalpy of formation of various metal hydrides;^50^ (d) schematic diagram describing continuous energy barriers in the process of hydrogen absorption (up) and illustration of the kinetic steps in the hydrogen storage process (down).^51^

The storage mechanism manifests through a complex three-phase equilibrium involving hydrogen gas, metallic magnesium, and magnesium hydride, with phase stability governed by the interplay of pressure, composition, and temperature (Fig. 3a). During isothermal hydrogenation, the process initiates with hydrogen dissolution in magnesium to form a solid solution (α-phase), characterized by the random occupation of interstitial sites within the hexagonal close-packed magnesium lattice. This process demonstrates unique crystallographic evolution, where hydrogen atoms systematically occupy tetrahedral sites, inducing localized lattice distortions that facilitate subsequent phase transformation. As hydrogen pressure increases, the system undergoes a pressure-dependent transformation to the hydride (β-phase) through nucleation and growth mechanisms. This α → β phase transition maintains constant pressure until complete conversion, following established phase equilibrium principles as evidenced by the characteristic plateau region in pressure–composition isotherms (Fig. 3b). The phase transformation involves substantial structural reorganization, generating significant internal stresses that influence subsequent reaction kinetics.

The thermodynamic behavior of this process can be quantitatively described through the van't Hoff relationship and molar Gibbs free energy equation:^52^2
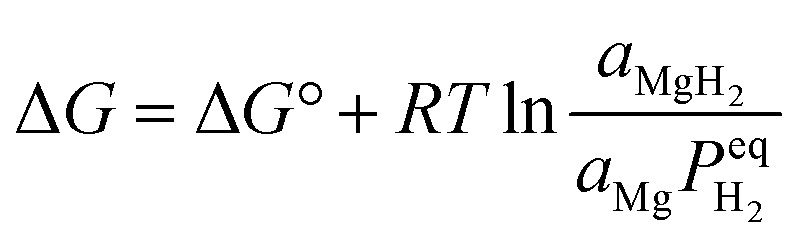
3
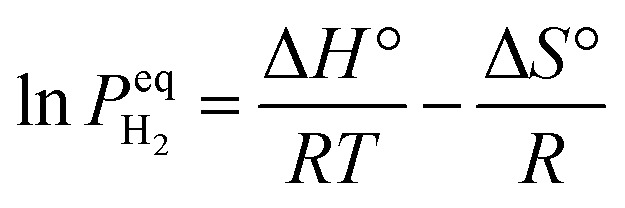
where 
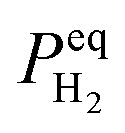
 represents the equilibrium hydrogen pressure, Δ*H*° and Δ*S*° denote standard enthalpy and entropy changes respectively, and activities (*a*) of solid phases approximate unity. The remarkable linearity between 
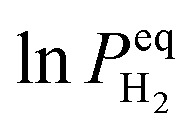
 and 
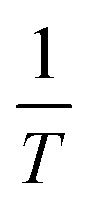
 enables precise determination of fundamental thermodynamic parameters, revealing Δ*H*° ≈ 74.7 kJ per mol H_2_ and Δ*S*° ≈ 130 J per (K mol) H_2_ for the Mg/MgH_2_ system. While Δ*S*° primarily reflects the transformation of molecular hydrogen gas to bound atomic hydrogen and remains relatively consistent across metal–hydrogen systems, Δ*H*° represents a key parameter for performance optimization. As illustrated in Fig. 3c, the formation enthalpies of various metal hydrides provide valuable guidance for thermodynamic tuning through compositional engineering. By alloying Mg with elements that form less stable hydrides, the operating temperature for hydrogen release can be significantly reduced while maintaining practical storage capacity. This thermodynamic framework establishes crucial design principles for interface engineering strategies aimed at optimizing both stability and reversibility.

The hydrogen storage mechanism involves distinct activation barriers that collectively determine the overall reaction kinetics (Fig. 3d). The complex energy landscape can be divided into several critical stages, each presenting specific kinetic challenges. The initial surface interaction stage, comprising physisorption (*E*_phys_ = 5–15 kJ per mol H_2_) and chemisorption (*E*_chem_ = 50–60 kJ per mol H_2_), establishes the crucial gas–solid interface. Following surface activation, the rate-determining step shifts to atomic hydrogen diffusion, exhibiting markedly different barriers in different phases: relatively facile diffusion in the α-phase (*E*_diff_ ≈ 21 kJ per mol H_2_) contrasts with substantially higher barriers in the β-phase (*E*_diff_ ≈ 250 kJ per mol H_2_). This transition in diffusion barriers coincides with the formation of a coherent hydride layer, known as the “blocking effect”, which becomes increasingly significant as the reaction progresses. The process kinetics follow the Arrhenius relationship:^39^4
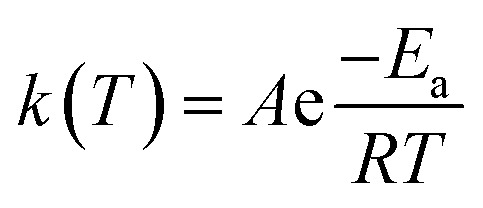
where the apparent activation energy (*E*_a_) reflects the dominant rate-limiting step under specific conditions. The exponential temperature dependence explains the requirement for elevated temperatures in practical applications, while the pre-exponential factor (*A*) relates to the frequency of attempted barrier crossings. This mechanistic understanding reveals that effective interface engineering must address both the surface activation barriers and bulk diffusion limitations simultaneously.

A particularly critical aspect in the reaction dynamics lies in the evolution of interfacial phenomena during hydrogen cycling. The formation of a coherent MgH_2_ surface layer creates a significant diffusion barrier that progressively impedes hydrogen transport to unreacted magnesium, known as the “blocking effect”.^53^ This self-limiting mechanism becomes increasingly dominant as the hydride layer thickens, manifesting through interface-controlled diffusion where the MgH_2_ layer impedes both hydrogen dissociation and atomic hydrogen diffusion. The severity of this blocking effect correlates strongly with the hydride layer thickness and morphology. Detailed microscopic studies have revealed that the interface between α-MgH_2_ and β-MgH_2_ phases plays a crucial role in hydrogen transport, where structural mismatches and accumulated strains can significantly affect local diffusion pathways. The transformation from α-MgH_2_ to β-MgH_2_ involves substantial structural reorganization, while the volume expansion during hydrogenation (∼32%) generates internal stresses that create additional kinetic barriers. Moreover, the formation of oxide layers or other surface impurities can further complicate these interfacial effects by introducing additional resistance to hydrogen transport.

These fundamental kinetic investigations reveal several critical principles for interface engineering in magnesium-based hydrogen storage systems. First, the multi-step nature of the storage process, with its distinct activation barriers, suggests that effective interface design must simultaneously address surface activation, hydrogen dissociation, and bulk diffusion. Second, the transition from surface-controlled to diffusion-controlled kinetics highlights the importance of maintaining accessible pathways for hydrogen transport throughout the reaction. The significant role of the “blocking effect” indicates that interface engineering strategies should focus on creating stable channels for hydrogen diffusion while preventing the formation of continuous barrier layers. Furthermore, the complex interplay between structural evolution and reaction kinetics emphasizes the need for interface designs that can accommodate volume changes while maintaining catalytic activity. These mechanistic insights guide the development of advanced interface architectures, from nanoscale catalytic sites to hierarchical diffusion networks, establishing the foundation for rational design of high-performance hydrogen storage materials.

## 3. Interface architecture through structure design

Extensive research has established nanostructuring as a transformative strategy for modulating both thermodynamic equilibria and reaction kinetics in the Mg/MgH_2_ system.^54,55^ This approach fundamentally alters the material's physicochemical properties through three primary mechanisms: quantum size effects that modify electronic structures and bonding energetics, enhanced surface phenomena that create abundant active sites for hydrogen interactions, and optimized interfacial architecture that facilitates mass transport.^56,57^ At the nanoscale, the dramatically increased specific surface area (typically exceeding 100 m^2^ g^−1^) creates an abundance of coordinatively unsaturated sites that serve as active centers for hydrogen dissociation and recombination. These sites exhibit distinct electronic configurations compared to bulk materials, leading to modified hydrogen binding energies and improved reaction pathways. Additionally, the nanoscale architecture introduces unique structural features that fundamentally alter hydrogen diffusion mechanisms, while the high density of engineered interfaces serves as preferential channels for hydrogen transport, effectively reducing kinetic barriers.^58^ The structure design of interface architecture—from one-dimensional confined channels to three-dimensional interconnected networks—enables precise tuning of interfacial structures, optimizing local chemical environments and mass transport pathways. This strategic approach systematically enhances hydrogen storage performance through rational interface design.

### 3.1. Free-standing nanostructures: size-dependent properties

Size-dependent properties of magnesium-based materials have emerged as a fundamental approach for modulating hydrogen storage performance through precise structural control at the nanoscale. Systematic investigations through both theoretical and experimental approaches have revealed that reducing particle size below critical dimensions (typically <10 nm) introduces significant modifications to the electronic band structure.^59,60^ This size-dependent electronic modulation directly impacts the strength of metal–hydrogen bonds, leading to systematic destabilization of both Mg and MgH_2_ phases. Most notably, when crystallite dimensions approach the quantum confinement regime (<2 nm), theoretical studies predict a dramatic reduction in formation enthalpy,^61^ effectively lowering the thermodynamic stability of the hydride phase. From a structural perspective, these size-dependent effects manifest through three primary mechanisms: quantum confinement phenomena that alter electronic band structures and bonding characteristics, dramatically enhanced surface-to-volume ratios that create abundant active sites for hydrogen dissociation, and modified interfacial energetics that influence both thermodynamic stability and reaction kinetics. These interrelated structural effects highlight the importance of precisely designing the architecture of Mg-based materials, ensuring that nanoscale dimensions are leveraged to overcome conventional limitations in hydrogen storage. Thus, structure design emerges as a critical tool in harnessing size-dependent effects, providing a rational pathway to develop high-performance Mg-based hydrogen storage systems with enhanced reversibility and tunable thermodynamic stability.

**Fig. 4:**
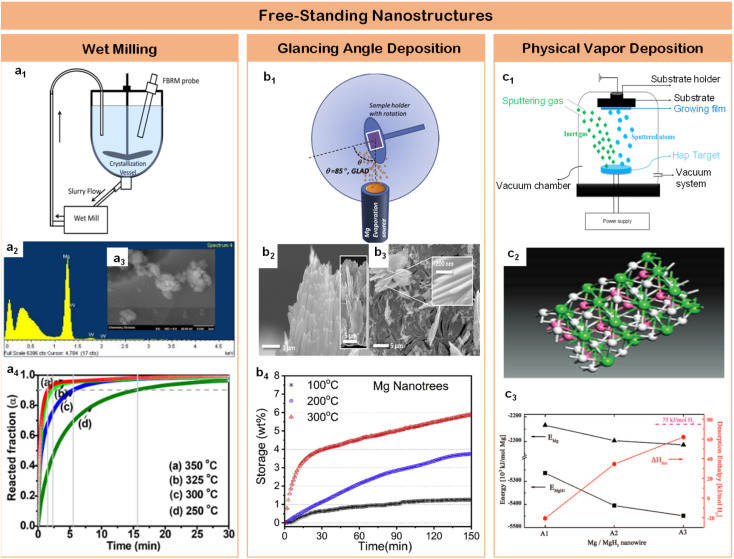
(a1) Glancing angle deposition (GLAD) method is illustrated; (a2) cross-sectional and top-view (a3) scanning electron microscopy (SEM) images of GLAD Mg nanotrees with stacks of nanoleaves grown on QCM substrates are shown; (a4) hydrogen absorption profiles of Mg nanotrees are shown as a function of time at 100, 200, and 300 °C under 30 bars of H_2_.^62^ (b1) Illustrate of wet mill; (b2) EDX of MgH_2_-8 h sample; (b3) SEM image of MgH_2_-8 h sample; (b4) MgH_2_-1 h and B, MgH_2_-8 h at different temperatures.^63^ (c1) Illustrate of physical vapor deposition; (c2) the structures of A3_MgH_2_ for before optimization; (c3) desorption enthalpy for MgH_2_ nanowires (red line) and the size effect of the calculated energies for magnesium (triangle) and MgH_2_ (tetragon) nanowires. The desorption enthalpy are normalized per mol of H_2_ released and the energies are normalized per Mg/MgH_2_ unit.^64^

Experimental validation of these size-dependent effects has progressed through systematic development of synthetic strategies and structural control methodologies. Banerjee *et al.*^62^ demonstrated a breakthrough in controlling particle size distributions through an innovative wet milling protocol (Fig. 4a1), achieving uniform crystallites (∼80 nm) with beneficial phase composition – notably the coexistence of γ-MgH_2_ and conventional α-MgH_2_ phases (Fig. 4a2 and a3). This dual-phase structure, combined with precise size control, resulted in exceptional kinetic performance: achieving 90% of theoretical capacity within remarkably short timeframes – 15.5, 5.5, 2.3, and 1.5 minutes at temperatures of 250 °C, 300 °C, 325 °C, and 350 °C respectively (Fig. 4a4). The systematic correlation between temperature and absorption rate highlights the critical role of size-controlled interfaces in enhancing hydrogen storage dynamics. The fundamental breakthrough in size control was achieved by Li *et al.*,^65^ who developed a vapor transport approach to synthesize magnesium nanostructures with controlled morphologies from basic nanospheres to complex sea-urchin architectures, establishing the critical relationship between particle morphology and hydrogen dissociation kinetics. Further sophistication in architectural design was demonstrated by Cansizoglu *et al.*^63^ through glancing angle deposition techniques, creating highly oriented magnesium “nanotrees” (Fig. 4b1–b3). These hierarchical structures combined the advantages of nanoscale dimensions with organized architecture, achieving 6.98 wt% H_2_ absorption at 300 °C while maintaining structural integrity through multiple cycles (Fig. 4b4). The evolution from GLAD techniques to physical vapor deposition (PVD) marked a significant advancement in dimensional control precision. This transition enabled unprecedented insights into size-property relationships in magnesium-based storage systems, establishing quantitative correlations between nanostructure dimensions and hydrogen storage behavior. The enhanced precision of PVD allowed for systematic investigation of interface effects across different size regimes, providing fundamental understanding of how dimensional confinement influences both thermodynamic and kinetic aspects of hydrogen storage. The ultimate refinement in dimensional control was achieved by Chen *et al.*,^64^ who synthesized free-standing magnesium nanowires with precisely controlled diameters (30–170 nm) through physical vapor deposition (Fig. 4c1). This precise control enabled unprecedented insights into size-dependent hydrogen storage behavior, revealing novel hydride phases including A1_MgH_3_ (Fig. 4c2), A2_MgH_2.33_, and A3_MgH_2.17_. To establish quantitative structure–property relationships, the desorption enthalpies of MgH_2_ nanowires were systematically analyzed by normalizing per mole of H_2_ released, with absolute energies standardized per mole of Mg (Fig. 4c3). This rigorous energetic analysis demonstrated clear dimensional dependence of thermodynamic properties. The smallest diameter nanowires (30–50 nm) exhibited optimal performance metrics, achieving 7.6 wt% absorption and 6.8 wt% desorption within 30 minutes at 100 °C, while larger diameter structures (150–170 nm) maintained respectable capacities of 6.5 wt% absorption and 5 wt% desorption under identical conditions. These comprehensive studies established clear correlations between dimensional confinement and hydrogen storage mechanisms.

However, when MgH_2_ particle dimensions approach the quantum confinement regime (<2 nm), the system exhibits dramatic changes in thermodynamic properties that challenge conventional understanding.^66^ While the size-dependent destabilization offers unprecedented opportunities for enhancing hydrogen storage performance, it simultaneously introduces significant challenges in maintaining structural integrity during practical applications. The exceptionally high surface energy of nano-magnesium hydride promotes aggressive particle agglomeration during hydrogen cycling, while thermal effects during absorption and desorption processes can lead to detrimental sintering that compromises the nanostructure. Even in systems demonstrating promising initial performance, the preservation of nanoscale dimensions through extended cycling remains a critical challenge. These fundamental limitations of free-standing nanostructures highlight the necessity for more sophisticated interface engineering strategies that can simultaneously address multiple aspects of hydrogen storage performance. The development of advanced confinement architectures, which can both stabilize nanoscale dimensions and maintain efficient hydrogen diffusion pathways, emerges as a promising direction for overcoming these challenges. This understanding naturally leads to the exploration of nanoconfinement strategies, where host materials can provide structural stabilization while preserving the advantages of nanoscale dimensions.

### 3.2. Nanoconfinement strategy: stabilized interface architecture

The challenge of maintaining nanostructured states through multiple hydrogen storage cycles has prompted the development of nanoconfinement strategies as a significant advancement in structural engineering. This approach addresses the fundamental limitations of free-standing nanoparticles by utilizing nanoporous host materials as structural scaffolds, effectively constraining magnesium-based materials while preventing particle agglomeration during thermal cycling. Through careful selection and engineering of host materials, nanoconfinement enables precise control over particle dimensions, interfacial interactions, and mass transport pathways, offering a comprehensive solution to the stability-performance trade-off in magnesium-based hydrogen storage systems. Interface architecture serves as a fundamental design principle in nanoconfinement strategies, by integrating structural stability with optimized interfacial properties, nanoconfinement offers a comprehensive approach to overcoming the long-standing stability-performance trade-off in magnesium-based hydrogen storage systems. The selection of appropriate host materials plays a crucial role in determining the effectiveness of nanoconfinement, with carbon-based materials emerging as particularly promising candidates due to their thermal stability, chemical inertness, and tunable pore architectures.

**Fig. 5:**
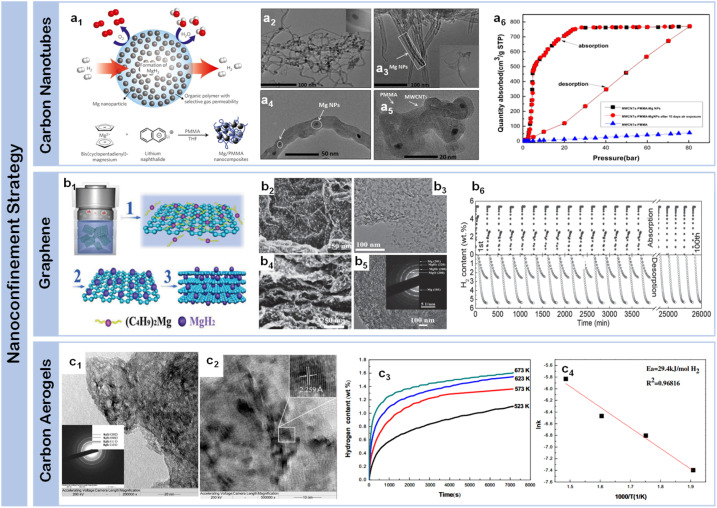
(a1) Schematic of hydrogen storage composite material: high-capacity Mg NCs are encapsulated by a selectively gas-permeable polymer and synthetic approach to formation of Mg NCs/PMMA nanocomposites; synthesis mechanism of MPMC TEM micrograph of MWCNTs-Mg NPs composite (a2), and MPMC (a3–a5); (a6) isothermal H_2_ auantity adsorbed of MPMC (0 day and 10 days exposure to air) and MWCNTs-PMMA assembles with various pressure using HPVA-II.^70^ (b1) Schematic illustration of the self-assembly of MgH_2_ NPs on GR; (b2) SEM (b3 and b4) and (b5) HRTEM images of MHGH-60; (b6) hydrogenation and reversible H_2_ absorption (under 30 atm hydrogen pressure) and desorption (under 0.01 atm hydrogen pressure) of and MHGH-75 at 200 °C.^71^ (c1) HRTEM micrographs and the corresponding SAED pattern (the inset of a) of MgH_2_-CA composite, a magnified view (c2) to show the size of dark region and the inset to display one of the darker regions with MgH_2_ lattice fringe; (c3) hydrogen absorption curves of Mg-CA composites measured at different temperatures and (c4) the corresponding ln *k*e1000/*T* plot.^72^

Carbon nanotubes (CNTs) have emerged as pioneering scaffold materials for magnesium-based hydrogen storage systems, offering unique dimensional advantages through their well-defined tubular architecture. The inherent one-dimensional structure of CNTs enables precise control over spatial confinement while providing robust mechanical stability during cycling processes, addressing a fundamental challenge in nanostructured systems. Wang *et al.*^67^ pioneered the integration of single-walled carbon nanotubes (SWCNTs) with magnesium hydride, demonstrating that SWCNTs could form interconnected networks during ball milling, creating efficient channels for hydrogen diffusion while constraining metal hydrides. This concept was significantly advanced by Amirkhiz *et al.*,^68^ who developed MgH_2_-SWCNTs composites that exhibited remarkably improved kinetics and maintained hydrogen absorption capacity through more than 100 cycles, highlighting the importance of stable network structures in long-term cycling performance. The optimization of nanotube dimensions has achieved enhanced control over magnesium hydride confinement, where engineered CNT frameworks create interconnected networks that facilitate both spatial organization and directional mass transport. This dimensional control strategy effectively prevents particle agglomeration while maintaining efficient hydrogen diffusion pathways, Cai *et al.*^69^ systematically investigated the influence of CNT content through an innovative co-deposition method utilizing aligned carbon nanotubes as a three-dimensional framework. By carefully controlling the milling time of Mg powder with multi-walled carbon nanotubes (MWCNTs) (denoted as MCX, where X represents stirring duration), they revealed that while CNTs enhanced grain boundary area and promoted nucleation, excessive CNT content could suppress nucleation by separating adjacent MgH_2_ particles, suggesting an optimal balance in the “chain” nucleation mechanism. A significant advance in dimensional organization emerged through Liang *et al.*'s development^70^ of a hybrid templating strategy incorporating MWCNTs into PMMA networks (Fig. 5a1). Their precise structural characterization revealed uniformly distributed MgH_2_ nanoparticles (3.6 ± 0.2 nm) (Fig. 5a2–a5), enabling exceptional hydrogen uptake of 6.7 wt% at 20 bar H_2_ and 200 °C that approaches the theoretical capacity limit (Fig. 5a6). The system's success stems from the hierarchical architecture's ability to simultaneously maintain spatial confinement while preserving efficient diffusion pathways. This controlled integration of polymer matrices and carbon nanotubes establishes a new paradigm for achieving stable nanoconfinement in magnesium-based systems. These systematic studies of CNT-based architectures demonstrate the critical role of structural organization in optimizing both dimensional confinement and mass transport capabilities.

Graphene have emerged as another class of effective scaffold materials for magnesium hydride nanoconfinement, offering unique advantages through their two-dimensional layered structure and excellent thermal conductivity. Zhang *et al.*^73^ developed a sophisticated approach to synthesize MgH_2_ nanoparticles uniformly supported on graphene surfaces through systematic control of nucleation and growth processes. Similarly, Zhang *et al.*^74^ obtained graphene-supported homogeneous MgH_2_ nanoparticles (MgH_2_/G) with an average particle size of 8.5 nm were synthesized *via* hydrogenation-induced self-assembly, which exhibiting a capacity retention of approximately 99.8% after five cycles. This concept was significantly advanced by Xia *et al.*,^71^ who demonstrated bottom-up assembly of monodisperse MgH_2_ nanoparticles on graphene (Fig. 5b1). Their systematic study revealed the formation of high-density MgH_2_ nanoparticles homogeneously distributed throughout both the surface and interlayer regions of graphene sheets (Fig. 5b2–b5), achieving 4.3 wt% hydrogen absorption within 60 minutes, representing 75.4% of the theoretical capacity (5.7 wt%). Notably, the system maintained robust cycling stability with over 98.4% capacity retention through 30 cycles (Fig. 5b6), attributed to the synergistic effects of graphene's mechanical support and enhanced interfacial charge transfer. Wang *et al.*^75^ further advanced this strategy by incorporating magnesium borohydride into the interlayer spaces of graphene, achieving unprecedented hydrogen storage capacity (12.0 wt%) while substantially reducing the dehydrogenation temperature by 190.5 °C compared to pristine materials. An innovative approach was demonstrated by Song *et al.*,^76^ who utilized the unique molecular structure of bituminous coal during thermal decomposition, creating an *in situ* formed partially graphitized carbon framework through combined ball milling and heat treatment. This dual-functional design not only provided spatial confinement but also contributed to hydrogen storage through a synergistic dual-mode mechanism, where the confined magnesium served as the primary storage medium while the carbonaceous framework simultaneously acted as both a structural stabilizer and a supplementary storage component.

Carbon aerogels represent an alternative approach in dimensional control strategies, offering distinct structural advantages through their hierarchical pore architecture. Their high surface area (>1000 m^2^ g^−1^), coupled with tunable pore dimensions, provides a versatile platform for investigating fundamental correlations between confined spaces and hydrogen storage dynamics. Early breakthrough came from Krijn P. de Jong *et al.*,^77^ who successfully incorporated magnesium into nanoporous carbon materials through molten infiltration, realizing ultrafine magnesium particles of 2–5 nm. However, this initial approach faced inherent limitations: poor wettability between molten magnesium and carbon materials coupled with high oxidation susceptibility significantly restricted the effective loading.^78^ A transformative advance came from Craig M. Jensen *et al.*,^79^ who developed an innovative solution-based approach using dibutyl magnesium (Bu_2_Mg) infiltration into carbon aerogels, followed by controlled high-pressure hydrogenation to form MgH_2_ within the nanopores. This methodology not only significantly improved loading capacity but also enabled precise control over particle size distribution. Nielsen *et al.*^80^ further refined this strategy, demonstrating that *in situ* hydride formation effectively prevented agglomeration while increasing hydride loading rates through carefully orchestrated nucleation and growth processes. Liu *et al.*^72^ made substantial progress in elucidating structure–property relationships in carbon aerogel confined systems, achieving MgH_2_ crystallites averaging 19.3 nm (Fig. 5c1 and c2). It can be found that the composite can absorb 1.10, 1.36, 1.55 and 1.61 wt% of hydrogen after 2 h at 250, 300, 350 and 400 °C, respectively (Fig. 5c3). Their detailed pressure–composition–temperature measurements revealed significant improvements in thermodynamic properties, with reduced hydrogenation and dehydrogenation enthalpies (65.1 ± 1.56 kJ per mol H_2_ and 68.8 ± 1.03 kJ per mol H_2_ respectively) compared to bulk magnesium. Most notably, the apparent activation energy for hydrogen absorption decreased to 29.4 kJ per mol H_2_ (Fig. 5c4), marking a dramatic improvement over micron-sized Mg particles. These enhancements stem from the dual functionality of carbon aerogels: providing numerous isolated nucleation sites for Mg/MgH_2_ formation while effectively preventing particle growth through spatial confinement.^81^

Building on the success of conventional carbon scaffolds, advanced carbonaceous architectures have emerged as transformative platforms for magnesium confinement, offering unique advantages through their diverse structural features and tunable surface chemistry. A transformative breakthrough in ordered porosity engineering came from Jia *et al.*,^82^ who developed a templated synthesis strategy for ordered mesoporous carbon (OMC) matrices. By precisely controlling the pore hierarchy and surface chemistry, they achieved significantly higher magnesium loading capacities compared to conventional carbon aerogels while maintaining nanoscale confinement. The ordered pore channels facilitated rapid hydrogen diffusion while the optimized surface functionality promoted uniform magnesium nucleation and growth. Konarova *et al.*^83^ further advanced the field through systematic investigation of pore filling dynamics in CMK-3 frameworks. Their groundbreaking work revealed a previously undiscovered threshold effect in nanoconfinement: optimal hydrogen storage performance was achieved only when the magnesium loading precisely matched the available pore volume, with excess loading disrupting the beneficial confinement effects. Zhao-Karger *et al.*^84^ achieved significant improvements through precise pore engineering, employing Bu_2_Mg as a magnesium precursor to infiltrate MgH_2_ into activated carbon fibers with sub-3 nm pores. This extreme size confinement led to a dramatic reduction in dehydrogenation activation energy from 195.3 ± 10 kJ mol^−1^ to 142.8 ± 2 kJ mol^−1^ compared to bulk materials. Shinde *et al.*^85^ developed self-assembled magnesium hydride embedded in three-dimensional activated carbon frameworks, where the hierarchically porous structure enabled effective confinement of MgH_2_ while maintaining rapid hydrogen diffusion pathways. Their system demonstrated exceptional performance metrics, achieving 6.6 wt% hydrogen absorption within 10 minutes at 150 °C and maintaining remarkable stability through 100 cycles. These advancements in carbon-based confinement strategies established fundamental principles for optimizing both spatial organization and mass transport in nanoconfined systems. While carbon-based materials excel in providing confinement effects with additional functional benefits, alternative hard templating approaches focusing purely on structural control have also emerged as an important strategy.

Beyond carbon-based materials, alternative templating strategies for precise structural control have emerged as promising approaches in magnesium confinement. Exploring ordered porous oxide templates, Cui *et al.*^86^ conducted systematic investigations of magnesium infiltration within anodic aluminum oxide (AAO) nanochannels. Their work revealed complex interfacial phenomena, including preferential nucleation along pore walls and the formation of beneficial secondary phases (MgO and Mg_17_Al_12_) that enhance cycling stability. Rather than hindering performance, these phase transformations created additional active sites while maintaining rapid hydrogen release capabilities over multiple cycles. Further advancing confinement strategies, Jeon *et al.*^87^ pioneered the development of gas-selective polymer matrices. By precisely engineering the pore structure and surface chemistry of PMMA templates, they achieved uniform dispersion of spherical magnesium nanocrystals (4.9 ± 2.1 nm) that exhibited excellent hydrogen storage capacity without requiring noble metal catalysts. This approach demonstrates the potential of rationally designed templates to simultaneously address particle size control and cycling stability, while offering unique opportunities for interface optimization through varied chemical compositions and coordination environments. These developments in alternative templating materials complement carbon-based systems by providing additional pathways for tuning interfacial interactions and mass transport properties, though challenges remain in achieving comprehensive spatial confinement for practical applications.

The systematic investigation of nanoconfinement strategies has revealed distinct advantages and limitations across different host materials. Carbon-based frameworks excel in providing confinement effects with additional functional benefits such as enhanced thermal conductivity and electron transfer, while ordered templates enable precise control over pore architecture and interface chemistry. However, optimizing the balance between high magnesium loading (>80 wt%) and interface stability remains a significant challenge, particularly in maintaining uniform dispersion and structural integrity during extended cycling. The development of scalable synthesis methods for sophisticated architectures faces inherent challenges in reproducibility and cost-effectiveness. These fundamental limitations in nanoscale confinement highlight the necessity for exploring more comprehensive approaches that can simultaneously address multiple aspects of hydrogen storage performance, naturally leading to the investigation of interface chemistry through compositional modification and multi-component design.

## 4. Interface chemistry *via* compositional modulation

Interface chemistry through compositional modulation represents a complementary strategy to interface architecture for advancing magnesium-based hydrogen storage systems.^88^ While structural design has demonstrated significant potential in enhancing performance, a fundamental electronic limitation inherent to magnesium – the lack of d-orbital electrons for efficient hydrogen activation – cannot be fully addressed through architectural design alone.^89–92^ This intrinsic electronic barrier necessitates strategic incorporation of catalytic components, particularly transition metals and electronically modified materials, to facilitate hydrogen dissociation and diffusion through modified interfacial electronic structures. The versatility of compositional engineering stems from multiple available mechanisms: from direct orbital-mediated hydrogen activation by transition metals to enhanced charge transfer through conductive networks. Moreover, the relative abundance and cost-effectiveness of these materials, especially compared to noble metal catalysts, make them particularly attractive for practical large-scale implementation in hydrogen storage systems.

### 4.1. Monometallic catalytic interfaces: mechanistic insights

Transition metals, characterized by their partially filled d-orbitals, have emerged as primary candidates for interface engineering in magnesium-based hydrogen storage through their unique ability to facilitate hydrogen dissociation.^93^ Their electronic configuration enables efficient orbital overlap with H_2_ molecules, creating lower energy pathways for H–H bond breaking through the formation of metal–hydrogen intermediates. The versatility of transition metals in this role stems from their ability to both accept electrons from H_2_ σ bonding orbitals and donate electrons back to σ* antibonding orbitals.^89,94–97^ Furthermore, their relatively high abundance and cost-effectiveness compared to noble metals make them particularly attractive for practical applications. The systematic investigation of monometallic systems has established fundamental correlations between electronic structure and catalytic activity, providing crucial guidance for developing more sophisticated multi-component catalytic interfaces.

**Fig. 6:**
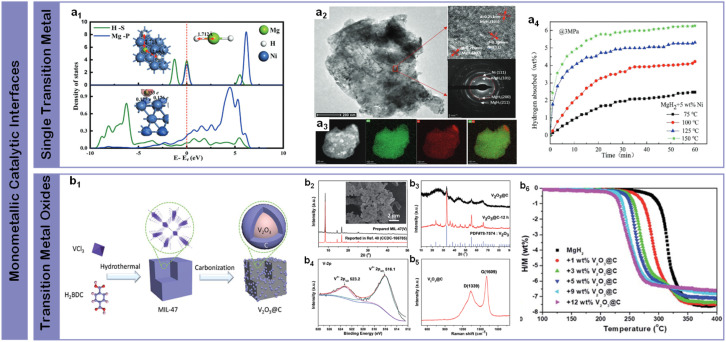
(a1) The adsorption configuration and corresponding morphological density of MgH_2_ on the Ni (111) surface; (a2) a TEM image with a HRTEM image and SAED pattern of MgH_2_ + 5 wt% Ni; (a3) the EDS pattern of MgH_2_ + 5 wt% Ni; (a4) isothermal hydrogen absorption curves at different temperatures of MgH_2_ + 5 wt% Ni.^99^ (b1) Schematic illustration of the preparation process of the V_2_O_3_@C nanocomposite; (b2) XRD patterns of the prepared MIL-47(V) and (b3) nano-V_2_O_3_@C (before and after heating), along with the inset SEM images of MIL-47(V), (b4) V 2p XPS spectra, and (b5) Raman spectra of nano-V_2_O_3_@C; (b6) MgH_2_-*x* wt% V_2_O_3_@C composites (*x* = 0, 1, 3, 5, 9, 12) volumetric hydrogen release curves for the same series.^100^

Single transition metal catalysts have revolutionized magnesium-based hydrogen storage through their distinctive electronic configurations and orbital-mediated interactions. Their intrinsic ability to facilitate hydrogen dissociation through d-orbital interactions while mediating electron transfer processes has established them as essential components for enhancing reaction kinetics at reduced temperatures. Initial breakthroughs came from Bassetti *et al.*^98^ through incorporation of 10 wt% Fe through high-energy ball milling, enabling hydrogen release of approximately 5 wt% within 600 s at 300 °C under 1 bar H_2_, establishing the fundamental viability of metal-based interface modification. A significant advance in reaction mechanism understanding emerged through Yang *et al.*'s^99^ comprehensive studies of nickel catalysis (Fig. 6a1), where uniformly distributed Ni nanoparticles throughout MgH_2_-5 wt% Ni composite achieved rapid dehydrogenation of 6.7 wt% within 3 minutes at 300 °C and subsequent absorption of 4.6 wt% H_2_ within 20 min at 125 °C (Fig. 6a4). The enhanced kinetics was attributed to the formation of beneficial Mg_2_Ni/Mg_2_NiH_4_ interfaces during cycling and as a superior “hydrogen pump”, enabling a more kinetically favorable pathway for hydrogenation and dehydrogenation processes, significantly modifying local bonding environments to facilitate hydrogen diffusion (Fig. 6a2 and a3).^101^ Further optimization through surface chemistry control was demonstrated by Yu *et al.*^102^ through precise ball-milling protocols incorporating commercial Zn nanoparticles, achieving improved kinetics and cycling reversibility through optimized interfacial contact. The versatility of chemical state modulation was highlighted by Sun *et al.*,^103^ who showed that combining MnCl_2_ and Mn particles could reduce the initial dehydrogenation temperature to 183 °C while enabling rapid absorption (3.0 wt% within 30 minutes at 100 °C) through complementary chemical interactions. These systematic investigations of single-metal catalysis have established critical correlations between catalyst distribution, chemical state, and hydrogen storage performance, while also revealing the inherent limitations of monometallic systems in simultaneously optimizing multiple performance metrics – a challenge that naturally leads to exploration of more sophisticated multi-component catalytic designs.

In contrast to pure metals, transition metal oxides introduce unique interfacial characteristics through coordinated metal–oxygen environments. The synergistic combination of structural stability from metal–oxygen frameworks and catalytic activity from coordinatively unsaturated metal sites provides additional pathways for optimizing hydrogen–metal interactions. Building on these fundamental studies of metal-based interfaces, Zhang *et al.*^104^ prepared MgH_2_–Nb_2_O_5_ nanocomposite which absorbed ∼4.4 wt% H_2_ within 60 min at 50 °C through uniform dispersion of Nb nanoparticles on MgH_2_ surfaces. Song *et al.*^105^ initiated systematic studies of magnesium–oxide interfaces through mechanical grinding under H_2_ (reactive mechanical grinding) with Cr_2_O_3_ and Al_2_O_3_, where Mg-10 wt% Cr_2_O_3_ achieved 5.87 wt% H_2_ absorption at 300 °C under 11 bar H_2_ within 60 min at the first cycle, while Mg-10 wt% Al_2_O_3_ absorbed 4.02 wt% H_2_ under identical conditions within 10 min, demonstrating the significant impact of oxide composition on interface reactivity. Further advancing the understanding of metal oxide interfaces, Gao *et al.*^106^ developed CoB/CNTs nanocomposites using CoCl_2_, NaBH_4_, and CNTs as precursors, achieving reduced onset and peak desorption temperatures of 214 °C and 271 °C respectively, with hydrogen release reaching 5.5 wt% within 19 minutes at 300 °C. Mechanistic studies revealed the formation of Co_3_MgC during dehydrogenation, which provided additional reaction sites and diffusion channels while maintaining stability through cycling. A significant breakthrough in oxide interface design came from Wang *et al.*,^100^ who synthesized V_2_O_3_@C nanocomposites through a simple two-step process (Fig. 6b1). Their detailed characterization revealed well-defined crystalline phases with distinct V^3+^ electronic states (Fig. 6b2–b5), enabling approximately 6.4 wt% hydrogen release within 20 minutes at 275 °C (Fig. 6b6), with remarkable stability of the metallic V phase through subsequent cycles. These systematic studies of metal oxide catalysis demonstrate the potential of controlled oxidation states and coordination environments in enhancing hydrogen storage performance, while highlighting the importance of stabilizing active phases through strategic interface design – insights that prove crucial for developing more sophisticated multi-component catalytic systems.

These systematic investigations of single-metal-based interfaces – from pure metals to metal oxides – have established fundamental principles for rational catalyst design in magnesium-based hydrogen storage materials. The progression from simple metal doping to sophisticated oxide architectures reveals both the complexity and opportunity in interface chemistry modification. While transition metals demonstrate direct catalytic effects through orbital-mediated hydrogen activation, their oxides offer additional functionalities through varied coordination environments and electronic states. However, the inherent limitations of single-component systems – particularly the trade-off between catalytic activity and long-term stability – naturally lead to the exploration of bimetallic interfaces that can potentially combine complementary properties through strategic compositional design. The insights gained from understanding single-metal interfaces, especially the critical roles of electronic structure and chemical bonding environments, provide crucial guidance for developing more sophisticated multi-component catalytic systems.

### 4.2. Bimetallic interface engineering: synergistic catalysis

Building on single-metal achievements, bimetallic systems have emerged as a superior strategy for enhancing magnesium-based hydrogen storage through sophisticated cooperative effects. By rationally combining transition metals with complementary electronic structures and catalytic functionalities, these systems enable simultaneous optimization of multiple performance aspects – from hydrogen dissociation kinetics to structural stability. The strategic selection and precise control of metal combinations create synergistic interfaces that significantly outperform single-component catalysts, while maintaining long-term cycling stability through controlled phase evolution and interface chemistry.

**Fig. 7:**
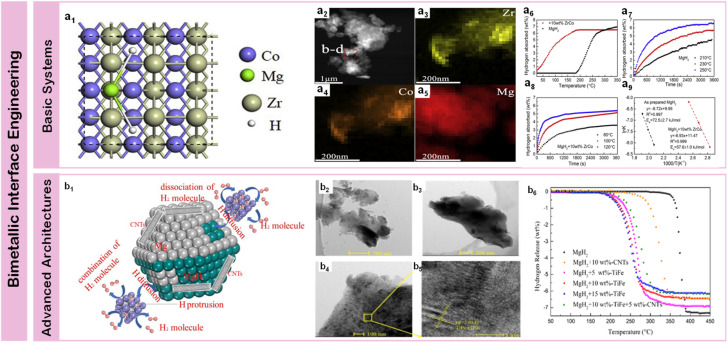
(a1) The MgH_2_ absorption on ZrCo (110); (a2) STEM-HAADF image and corresponding EDS mapping (a3–a5) of the MgH_2_ + 10 wt% ZrCo composite; (a6) non-isothermal hydrogenation curves; (a7 and a8) isothermal hydrogenation curves; (a9) and the corresponding Arrhenius plots of MgH_2_ with and without 10 wt% ZrCo.^108^ (b1) Schematic summary of catalytic mechanism for the TiFe and CNTs catalyzed MgH_2_ particles; (b2) TEM images and (b3–b5) corresponding HRTEM image of as-prepared TiFe; (b6) non-isothermal desorption curves of MgH_2_/TiFe, MgH_2_/CNTs and MgH_2_/TiFe/CNTs composites.^109^

Early explorations of bimetallic systems revealed that strategic combinations of transition metals could create unique synergistic effects through complementary electronic interactions and modified interfacial chemistry. A significant advance in kinetic enhancement was achieved by Yang *et al.*^107^ through precisely engineered FeCo nanosheets (50 nm thickness), where the synergistic coupling between Fe and Co d-orbitals enabled exceptional performance – achieving 6.7 wt% hydrogen absorption within one minute at 300 °C while initiating dehydrogenation at 200 °C. Compared to Fe-based systems, Zr exhibits enhanced catalytic performance through its electronic configuration (4d^2^5s^2^), enabling more efficient hydrogen activation while forming thermodynamically favorable intermetallic phases that reduce desorption temperatures through modified binding energies. Zhang *et al.*'s^108^ development of ZrCo composites (Fig. 7a1), featuring uniform particle distribution (∼500 nm) with homogeneous elemental dispersion (Fig. 7a2–a5). Their rationally designed ZrCo nanosheets demonstrated remarkable bifunctionality, where Zr facilitated hydrogen dissociation while Co enhanced diffusion kinetics, enabling absorption of 4.4 wt% H_2_ within 10 minutes at 120 °C under 3 MPa pressure (Fig. 7a6–a9). Further optimization of low-temperature performance came through Zhu *et al.*'s^110^ ZrMn_2_ nanoparticles further advanced this concept by enabling hydrogen release initiation at 181.9 °C – approximately 160 °C lower than pristine MgH_2_, with exceptional kinetics of 6.7 wt% desorption within 5 minutes at 300 °C and rapid uptake of 5.3 wt% hydrogen at 100 °C within 10 minutes. In the progression of transition metal catalysis, Ti presents distinct advantages through its electronic structure and atomic properties. The combination of lower atomic mass and favorable d-orbital occupancy enables the formation of hydrides with reduced formation enthalpies (Δ*H*_f_), resulting in accelerated sorption kinetics. Moreover, Ti's electronic configuration facilitates synergistic interactions with secondary transition metals (Mn, V, Ni), creating cooperative catalytic effects. Initial breakthroughs came from El-Eskandarany *et al.*^111^ through uniform incorporation of TiMn_2_ spherical particles (100–320 nm), where the MgH_2_-10 wt% TiMn_2_ nanocomposite demonstrated both rapid kinetics (5.1 wt% H_2_ absorption within 100 s under 10 bar pressure) and exceptional cycling durability through 600 hours at 225 °C, establishing fundamental principles for stable metal–metal interfaces. While both Mn and V form stable intermetallic compounds through similar electronic configurations, V demonstrates superior catalytic performance due to its partially filled d-orbitals, which provide additional electron transfer pathways for enhanced hydrogen dissociation and diffusion kinetics. Comprehensive performance enhancement was achieved through multi-component design, exemplified by Liu *et al.*'s^112^ Mg-9.6 wt%Ti-2.9 wt%V nanocomposites synthesized *via* plasma-metal reactions, achieving substantially reduced activation energies (29.2 and 73.8 kJ mol^−1^ for hydrogenation and dehydrogenation). Building on this concept, their team extended the investigation to Ti–Ni systems,^113^ where MgH_2_-10 wt% Ti_2_Ni cold-rolled composites achieved optimized absorption kinetics (5.7 wt% H_2_ within 400 s) with reduced activation energy of 87.3 kJ mol^−1^ at 225 °C, highlighting the importance of processing methods in interface engineering. These systematic investigations of basic bimetallic systems established fundamental principles for catalyst design – demonstrating that strategic combinations of transition metals could achieve synergistic effects through complementary electronic structures and optimized interfacial interactions, while maintaining long-term stability through controlled phase evolution.

Beyond simple bimetallic combinations, composite architectures incorporating multiple metallic components enable systematic tuning of electronic structures and interfacial interactions, establishing new pathways for enhancing magnesium-based hydrogen storage performance. Through rational design of component distribution and local chemical environments, these advanced systems achieve unprecedented control over reaction mechanisms. A significant breakthrough came from Lu *et al.*'s^109^ TiFe-CNTs system (Fig. 7b1), where precisely controlled particle distribution (∼500 nm) with clear lattice correlation to TiFe (110) planes (Fig. 7b2–b5) achieved 6.5 wt% hydrogen release within 10 minutes at 300 °C (Fig. 7b6) through enhanced charge transfer pathways. This concept was further advanced by Duan *et al.*^114^ through sophisticated Co@Pd-carbon nanotubes composites, achieving 7.30 wt% H_2_ release at 325 °C in just 8 minutes through combined effects of Co@Pd bimetallic synergy and *in situ* formed Mg_2_Co/Mg_6_Pd “hydrogen pump” phases. Pan *et al.*^115^ revealed the importance of controlled phase evolution in their Co_3_V_2_O_8_ nanosheets system, where decomposition into Co and V_2_O_3_ active species enabled rapid absorption of 4.87 wt% hydrogen within 1 minute at 150 °C through synergistic modification of H–H and Mg–H bonds. The strategic integration of bimetallic active sites with conductive carbon nanotube frameworks creates hierarchical architectures that optimize electron transfer processes and mass transport pathways, demonstrating the potential for comprehensive performance enhancement through multi-component design. Zhang *et al.*^116^ extended this strategy by developing Na_2_Ti_3_O_7_ through hydrothermal and solid-phase methods, with MgH_2_–Na_2_Ti_3_O_7_ composites demonstrating remarkable kinetics – releasing 6.5 wt% hydrogen within 6 and 16 minutes respectively at 300 °C. Complex transition metal oxides have emerged as particularly effective catalysts, exemplified by Huang *et al.*'s^117^ NiTiO_3_ and CoTiO_3_ systems achieving significantly reduced dehydrogenation temperature (235 °C) and enhanced rate (∼0.1842 wt% min^−1^). Chen *et al.*^118^ further advanced this approach through Ni/TiO_2_ nanocomposites (∼20 nm) prepared *via* solvothermal synthesis, enabling hydrogen desorption at 232–135.4 °C lower than ball-milled MgH_2_, while achieving 6.5 wt% release within 7 minutes at 265 °C and 5 wt% absorption within 10 minutes at 100 °C, attributed to the synergistic combination of multi-valence Ti electron channels and Mg_2_NiH_4_/Mg_2_Ni hydrogen pump interfaces. These sophisticated interface designs open new pathways for achieving comprehensive performance enhancements through cooperative catalytic effects.

These systematic investigations of bimetallic systems – from basic bimetallic combinations to advanced multi-component architectures – have established fundamental principles for rational interface design in magnesium-based hydrogen storage systems. The progression from simple metal–metal interfaces to sophisticated multi-level catalytic networks reveals both the complexity and opportunity in optimizing interfacial phenomena through strategic compositional and architectural engineering.

### 4.3. Multi-component interface design: entropy-driven strategies

Recent advances in multi-component alloy catalysis have demonstrated remarkable progress through systematic design of medium-entropy and high-entropy systems, where the synergistic integration of multiple transition metals creates unprecedented catalytic environments. The strategic combination of three or more metallic elements enables comprehensive optimization of hydrogen storage performance through complementary electronic structures and coordinated phase evolution. These advanced alloy systems represent a transformative strategy that integrates the advantages of binary catalysts while introducing additional cooperative effects through precisely controlled compositional complexity.

**Fig. 8:**
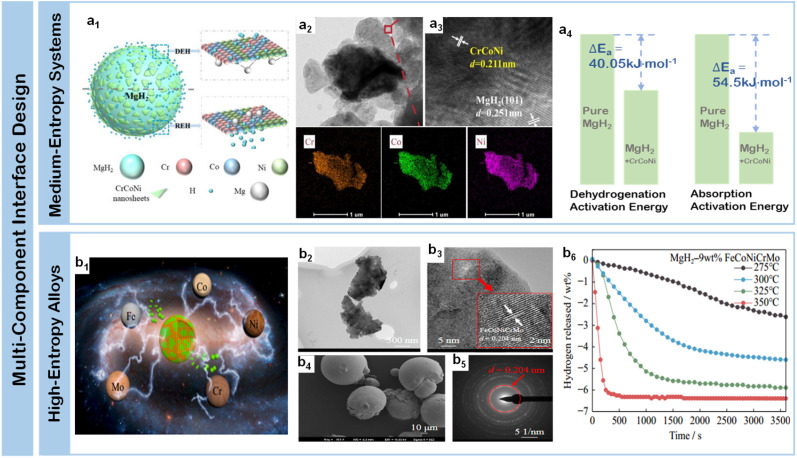
(a1) Mechanism diagram of dehydrogenation and rehydrogenation of the CrCoNi catalyzed MgH_2_/Mg system; (a2 and a3) HRTEM image and elemental mapping of MgH_2_ + 9 wt% CrCoNi; (a4) energy diagram for hydrogen absorption and desorption of MgH_2_ + 9 wt% CrCoNi.^119^ (b1) Mechanism diagram of dehydrogenation (DEH) and rehydrogenation (REH) of the FeCoNiCrMo catalyzed MgH_2_/Mg system; (b2) TEM and (b3) HRTEM of ball-milled FeCoNiCrMo; (b4) SEM of as-received FeCoNiCrMo; (b5) SAED of ball-milled FeCoNiCrMo; (b6) MgH_2_-9 wt% FeCoNiCrMo in isothermal mode.^120^

Medium-entropy alloy catalysts have emerged as effective platforms for enhancing magnesium-based hydrogen storage through sophisticated electronic modulation. Li *et al.*^119^ demonstrated the effectiveness of CrCoNi alloy catalyst (Fig. 8a1), where precise structural characterization revealed uniform lattice spacing (*d* = 0.204 nm) alongside MgH_2_ (101) planes (Fig. 8a2 and a3), achieving significant reduction in initial dehydrogenation temperature by 130 °C while releasing 4.84 wt% H_2_ within 5 minutes at 300 °C. The apparent activation energies were reduced by 45 and 55 kJ mol^−1^ for dehydrogenation and rehydrogenation respectively (Fig. 8a4), attributed to the cooperative electronic effects among Cr, Co, and Ni elements. Advancing this concept, Zhang *et al.*^121^ developed Zr–Ti–Co medium-entropy system integrated with carbon nanotubes, where the synergistic combination reduced the initial desorption temperature to 180 °C while achieving 90% hydrogen release within 10 minutes at 300 °C. The system demonstrated remarkably low activation energies of 70.5 ± 7.8 kJ mol^−1^ for dehydrogenation and 35.8 ± 3.8 kJ mol^−1^ for rehydrogenation through optimized interfacial charge transfer. These medium-entropy alloy studies demonstrate the emergence of collective interfacial effects beyond simple binary combinations – where the coordinated electronic interactions among multiple transition metals create unique local environments that simultaneously optimize hydrogen dissociation kinetics and mass transport, while maintaining structural stability through synergistic phase evolution.

Building on the insights from medium-entropy systems, high-entropy alloys represent a further advancement in multi-component interface engineering through the incorporation of five or more metallic elements, enabling unprecedented control over local electronic structures and reaction pathways. A significant breakthrough in high-entropy alloy catalysis was achieved by Gupta *et al.*,^122^ who developed a quaternary CuNiCoFe system with precisely controlled composition (Cu_0.15_Ni_0.35_Co_0.25_Fe_0.25_). This rationally designed multi-component catalyst enabled remarkable hydrogen storage performance: 6 wt% desorption at 250 °C coupled with rapid absorption kinetics reaching 3.5 wt% within 3 minutes. The enhanced performance was attributed to the synergistic electron transfer effects among the multiple metallic elements, demonstrating the advantages of increased configurational entropy in catalytic interfaces. Further advancing the high-entropy strategy, Zhong *et al.*^120^ developed a sophisticated quinary FeCoNiCrMo system that exemplifies the benefits of increased compositional complexity. Detailed structural characterization revealed a unique combination of transparent nanosheet morphology with well-defined lattice spacing (0.204 nm) (Fig. 8b2 and b3) and hierarchical spherical assemblies at the micron scale (Fig. 8b4 and b5). When integrated with MgH_2_ at an optimized loading of 9 wt%, this high-entropy catalyst demonstrated comprehensive performance enhancement: hydrogen release capacity of 5.89 wt% within 60 minutes at 200 °C and absorption capacity of 5.10 wt% within 30 minutes at 175 °C (Fig. 8b6). The exceptional kinetics can be attributed to the creation of numerous heterogeneous activation sites and interconnected diffusion channels through the cooperative effects among the five metallic elements (Fig. 8b1). This work demonstrates how increasing the number of metallic components can create more sophisticated catalytic environments through entropy-driven optimization of local electronic structures and atomic arrangements. Wan *et al.*^123^ advanced this strategy through FeCoNiCrMn high-entropy alloy that remarkable performance of 5.8 wt% H_2_ release within 4 minutes at 300 °C and 5.5 wt% H_2_ absorption within 0.5 minutes at 150 °C, demonstrating the potential of multi-component electronic modulation in promoting MgH_2_ decomposition. The remarkable performance achieved through high-entropy alloy catalysts establishes new principles for interface engineering – demonstrating that the strategic integration of multiple metallic components can create highly active and stable interfaces through cooperative electronic effects and controlled phase evolution, opening new possibilities for developing next-generation hydrogen storage materials.

The systematic investigation of interface effects in magnesium-based hydrogen storage systems – from transition metal catalysts to electronically modified carbon materials – has established fundamental principles for rational catalyst design and interface engineering. The progression from single-component to multi-component systems reveals both the complexity and opportunity in optimizing interfacial phenomena. While transition metals excel in providing direct catalytic pathways through orbital-mediated interactions, electronically modified support materials demonstrate complementary capabilities in charge transfer and structural stabilization. The synergistic integration of multiple functional components creates unprecedented opportunities for performance enhancement: reduced operating temperatures below 150 °C, practical absorption/desorption rates exceeding 6 wt% H_2_ within minutes, and exceptional cycling stability through extended operation. However, significant challenges remain in achieving practical impel mentation: the trade-off between catalytic loading and interface stability, the complexity of synthesizing sophisticated architectures at scale, and the need for mechanistic understanding of interface evolution during long-term cycling. These insights establish crucial design principles for developing next-generation hydrogen storage materials through sophisticated interface chemistry.

## 5. Synergistic integration of interface architecture and chemistry

Interface engineering represents a transformative strategy that integrates precise architectural control with strategic compositional design in magnesium-based hydrogen storage systems. While interface architecture through structural design and interface chemistry through compositional modulation have individually demonstrated notable improvements, the synergistic integration of both approaches enables unprecedented performance enhancements through multi-scale interface optimization. This comprehensive strategy simultaneously addresses multiple aspects of hydrogen storage performance: nanoconfinement provides structural stability and shortened diffusion paths, while *in situ* catalysis promotes reaction kinetics through optimized active sites. Recent advances in synthetic methodology and characterization techniques have enabled sophisticated control over both spatial organization and chemical composition at multiple length scales, creating hierarchical heterostructured materials with precisely engineered interfaces. This dual-functional approach has demonstrated remarkable success in simultaneously addressing key challenges of particle agglomeration and slow reaction kinetics through controlled spatial confinement while effectively catalyzing hydrogen sorption reactions, opening new pathways for performance optimization through synergistic effects.

### 5.1. One-dimensional hybrids: confined catalytic channels

Carbon nanotubes (CNTs) and electrospun nanofibers have emerged as archetypal one-dimensional platforms for hydrogen storage applications, owing to their unique combination of structural stability, high surface area, and excellent thermal/electrical conductivity. The integration of these one-dimensional materials with magnesium-based systems has demonstrated remarkable synergistic effects through multiple mechanisms: their tubular structure provides efficient hydrogen diffusion channels, while their high electrical conductivity facilitates charge transfer during hydrogen dissociation/recombination processes.^124–126^ Most importantly, their linear geometry enables sophisticated control over both catalyst distribution and spatial confinement at the nanoscale, creating hierarchical interfaces that optimize both thermodynamic and kinetic properties.

**Fig. 9:**
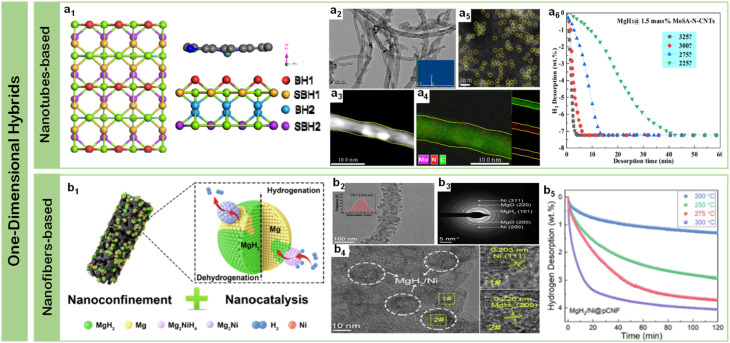
(a1) Top views of the MgH_2_ (110) surface and side view of the MgH_2_@MoSA-N-CNTs. White, green, gray, blue, and cyan spheres represent H, Mg, C, N, and Mo. Red, orange, blue and purple spheres respectively denote the first, second, third, and fourth layers of atomic H; (a2) TEM image of MoSA-N-CNTs; (a3) and (a4) dark-field scanning TEM and EDS mapping of MoSA-N-CNTs; (a5) HRTEM of MoSA-N-CNTs; (a6) isothermal dehydrogenation curves of MgH_2_@MoSA-N-CNTs and pure MgH_2_.^128^ (b1) Schematic diagram of the enhanced hydrogen storage performances of the MgH_2_/Ni@pCNF nanocomposites; (b2–b4) TEM observations of the as-synthesized MgH_2_/Ni@pCNF: (b5) isothermal dehydrogenation curves of MgH_2_/Ni@pCNF at different temperatures.^129^

SWCNTs have emerged as indispensable components in magnesium-based hydrogen storage systems, offering unique advantages through their well-defined molecular channels and exceptional electron transport properties. The strategic incorporation of SWCNTs has become a standardized approach for enhancing hydrogen storage performance, particularly in facilitating rapid hydrogen diffusion and maintaining structural integrity during cycling processes. A fundamental breakthrough in CNT-based systems came from Duan *et al.*,^127^ who developed MgH_2_@Ni-CNTs nanocomposites through strategic integration of catalytic doping and spatial confinement. This dual-functional design, where CNT frameworks provided structural stabilization while uniformly dispersed Ni nanoparticles enhanced reaction kinetics, lowered the dehydrogenation temperature to 250 °C, with hydrogen release of 7.29 wt% and absorption of 7.2 wt% at 200 °C within 30 minutes, maintaining enhanced performance through 10 cycles. A breakthrough in atomic-level catalyst design was achieved by Duan *et al.*,^128^ who pioneered single-atom catalysis by constructing an N-doped carbon nanotube supported Mo single-atom catalytic system (MoSA-N-CNTs) (Fig. 9a1). High-resolution characterization revealed ultrafine Mo atoms uniformly distributed on the hollow N-CNTs surface (Fig. 9a2–a4), with MgH_2_-1.5 wt% MoSA-N-CNTs generating 7.23 wt% H_2_ within 10 minutes through optimized atomic dispersion and electronic modification (Fig. 9a5). Further sophistication in CNT-based interfaces was demonstrated by Kajiwara *et al.*,^130^ who developed MgH_2_–Nb_2_O_5_-CNT composites, where the synergistic combination of oxide catalysis and CNT confinement enabled superior stability through extended cycling while maintaining rapid absorption kinetics. The evolution from single-walled to MWCNTs represents a significant advancement in interface engineering. MWCNTs exhibit superior mechanical robustness due to their concentric tubular architecture, while their increased surface area creates abundant active sites for hydrogen interactions. This hierarchical structure enables more sophisticated control over both spatial confinement and catalytic functionality, establishing new possibilities for enhanced hydrogen storage performance. Yuan *et al.*^131^ successfully incorporated uniformly dispersed 3 nm Pd nanoparticles onto MWCNT surfaces, with the resulting Mg_95_–Pd_3_/MWCNTs_2_ composite exhibiting exceptional kinetics – achieving 6.67 wt% hydrogen absorption within 100 s at 200 °C and 6.66 wt% desorption within 1200 s at 300 °C through enhanced interface contact area and improved thermal management. This progression in CNT-based interface engineering demonstrates the versatility of tubular architectures in simultaneously addressing multiple aspects of hydrogen storage performance: providing confined channels for hydrogen diffusion, maintaining structural integrity during cycling, and enabling precise catalyst distribution from atomic to nanoscale dimensions.

Electrospun nanofibers represent another class of one-dimensional architectures combining unique advantages of controllable morphology, tunable porosity, and versatile composition for sophisticated interface engineering in magnesium-based hydrogen storage. Early progress in this direction was demonstrated by Chen *et al.*,^132^ who developed a sequential process combining single-nozzle electrospinning with calcination and reduction to construct porous nanofibers integrating nickel active sites (50 nm diameter) within their structure. This integrated architectural design enabled remarkable hydrogen storage characteristics: dehydrogenation initiated at 143 °C with complete desorption (7.02 wt%) within 11 minutes at 325 °C, while the reduced activation energy of 81.5 kJ mol^−1^ reflected the synergistic effects of the fiber's confined geometry and uniformly distributed catalytic interfaces. A significant advancement in nanofiber engineering emerged through Ren *et al.*'s^129^ work on MOF-derived N-doped layered porous carbon nanofibers (pCNF) with integrated MgH_2_/Ni interfaces (Fig. 9b1). This system demonstrated precise structural hierarchy, featuring magnesium hydride domains (∼15 nm diameter) homogeneously distributed throughout the pCNF framework (Fig. 9b2), with selected area electron diffraction (SAED) patterns and HRTEM analyses revealing well-defined crystalline phases (Fig. 9b3 and b4). The hierarchical structure leveraged multiple synergistic effects: the pCNF framework provided spatial confinement, N-doping enhanced electron transfer, and the formed Mg_2_Ni/Mg_2_NiH_4_ interfaces facilitated hydrogen transport through a “pump” mechanism, ultimately achieving 4.1 wt% H_2_ absorption at 200 °C (Fig. 9b5) with remarkable stability – maintaining over 95.4% capacity through 10 cycles at 300 °C. These developments in electrospun nanofiber systems complement CNT-based approaches by offering greater flexibility in compositional design and hierarchical structure control, though optimizing the balance between fiber diameter, porosity, and catalytic loading remains crucial for practical applications.

One-dimensional hybrid architectures have demonstrated remarkable potential in integrating spatial confinement with interfacial catalysis through their unique linear geometries. Table 1 summarizes the hydrogen storage properties of one-dimensional nanomaterials, highlighting the effects of different structures (such as nanotubes and nanofibers) on rehydrogenation activation energy (*E*_ab_), dehydrogenation activation energy (*E*_de_), and cycling stability (almost no degradation after X cycles). CNT-based systems excel in providing well-defined channels for hydrogen diffusion and stable frameworks for catalyst anchoring, electrospun nanofibers offer greater flexibility in compositional design and hierarchical structure control. These complementary approaches establish fundamental principles for interface engineering at multiple length scales, from atomic-level catalyst distribution to microscale architecture organization. However, practical implementation faces common challenges in achieving optimal magnesium loading while maintaining uniform dispersion and long-term structural stability. These insights into one-dimensional confined catalytic channels provide valuable direction for exploring more complex two-dimensional architectures, where expanded interfacial areas offer new opportunities for enhanced catalytic interactions.

**Table 1:** Hydrogen storage properties of one-dimensional materials

Materials	Structure	*E*_ab_ (kJ per mol H_2_)	*E*_de_ (kJ per mol H_2_)	Cycling stability	Ref.
Ni-CNTs	Nanotube	—	74.8	10	127
MoSA-N-CNTs	Nanotube	33.97	68.43	—	128
Nb_2_O_5_-CNTs	Nanotube	—	—	20	130
Mg_95_–Pd_3_/MWCNTs_2_	Multi-walled nanotubes	—	78.6	—	131
Ni@nanofibers	Mesoporous nanofibers	—	81.5	—	132
Ni@pCNF	Porous nanofibers	25.4 ± 2.92	96.58 ± 4.16	10	129

### 5.2. Two-dimensional composites: interface-enhanced catalysis

Two-dimensional (2D) materials offer unique interfacial advantages for magnesium-based hydrogen storage through their distinctive structural characteristics: atomic-layer thickness, extensive surface area, and abundant active sites.^133–135^ Their planar architecture enables sophisticated interface engineering at the molecular level, where strategic modification of surface chemistry and electronic structure can significantly enhance both catalytic activity and structural stability.^136–138^ The layered configuration provides extensive contact interfaces for hydrogen dissociation while facilitating charge transfer processes essential for enhanced kinetics.

**Fig. 10:**
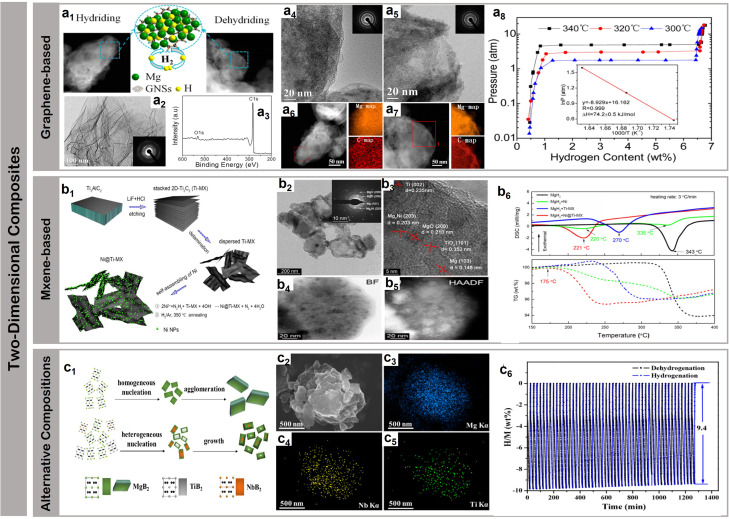
(a1) TEM image of GNSs; (a2) the inset of panel a is the SAED pattern of GNSs; (a3) survey XPS spectra of GNSs; TEM micrographs of the MgH_2_-5GNSs composites: (a4) before and (a5) after cyclic measurements; HAADF STEM micrographs and element mapping images of the MgH_2_-5GNSs composites: (a6) before and (a7) after cyclic measurements. Insets in panels a and b are selected area electron diffraction (SAED) patterns; (a8) pressure–composition–isothermal curves of the milled MgH_2_-5GNSs at different temperatures. The inset is the Van't Hoff plot of the milled MgH_2_-5GNSs derived from the dehydrogenation PCI measurements.^139^ (b1) Schematic illustrations of the synthetic strategy applied for the Ni@Ti-MX catalyst; (b2) bright-field TEM image and the corresponding SAED pattern (inset), (b3) HRTEM micrograph, and (b4 and b5) EDS mapping of the dehydrogenated MgH_2_ + Ni@Ti-MX composite; (b6) DSC and TG profiles of MgH_2_, MgH_2_ + Ni, MgH_2_ + TiMX, and MgH_2_ + Ni@Ti-MX, obtained at a heating rate of 3 °C min^−1^.^140^ (c1) Schematic illustration of the nucleation and growth processes of MgB_2_ in the LMBH-5TNO systems; (c2) SEM image of the 50th hydrogenated product of the LMBH-5TNO system and the corresponding EDS elemental mappings of (c3) Mg, (c4) Nb, and (c5) Ti; (c6) cycling curves of the LMBH-5TNO system under a regime of 400 °C/0.4 MPa/20 min for dehydrogenation and 350 °C/10 MPa/5 min for hydrogenation.^141^

Among two-dimensional materials, graphene-based architectures have established themselves as prime candidates for interface engineering in magnesium-based hydrogen storage systems, owing to their unique combination of atomic-level thickness, superior charge transport capabilities, and precisely controllable surface functionality. This exceptional versatility in both structural and electronic properties enable sophisticated manipulation of hydrogen storage interfaces at the molecular level. Early investigations by Liu *et al.*^139^ provided fundamental insights through systematic studies of few-layer, highly crumpled graphene nanosheets (GNS) through thermal exfoliation (Fig. 10a1–a3), where the engineered architectures maintained structural integrity through cycling with clear framework preservation (Fig. 10a4 and a5) and persistent fine grain distribution (Fig. 10a6 and a7), exhibiting characteristic thermodynamics of 74.2 ± 0.5 kJ per mol H_2_ from van't Hoff analysis (Fig. 10a8). Building on this foundation, Cho *et al.*^142^ advanced the field through strategic heteroatom doping, where both B900-rGO-Mg and N700-rGO-Mg systems demonstrated enhanced kinetic performance through modified interfacial interactions while maintaining bulk thermodynamic properties, revealing the predominant kinetic enhancement mechanism of graphene-based interfaces. Significant breakthroughs in performance came through the integration of catalytic metals with graphene frameworks. Cho ES *et al.*^143^ demonstrated this through nickel-modified graphene oxide frameworks supporting magnesium nanocrystals, achieving 6.5 wt% H_2_ capacity with sustained cycling stability through 30 cycles, while notably reducing desorption and absorption enthalpies to 66.9 and 63.9 kJ per mol H_2_, respectively. Further advancing this concept, Ji *et al.*^144^ developed sophisticated Fe–Ni/rGO architectures that achieved exceptional kinetics – enabling hydrogen uptake of 5.4 wt% within 20 minutes at 125 °C and 5.0 wt% at 100 °C with reduced activation energies of 42.3 ± 3.3 kJ mol^−1^, maintaining remarkable stability through 50 cycles at 6.9 wt% capacity through synergistic effects between Mg_2_Ni/Mg_2_NiH_4_ interfaces and the conductive graphene framework. These progressive advances demonstrate the exceptional potential of graphene-based materials in simultaneously addressing multiple performance aspects through interface engineering: providing efficient electron transport pathways, maintaining structural stability, and enabling precise control of interfacial chemistry, though challenges remain in optimizing magnesium loading while preserving uniform dispersion for practical applications.

MXenes represent a revolutionary class of two-dimensional materials synthesized through selective etching of ‘A’ elements (typically Al or Si) from MAX phase precursors – layered ternary carbides or nitrides. Unlike graphene's monolayer carbon structure, MXenes feature an intrinsically multilayered architecture that provides enhanced surface area and catalytically active sites through the incorporation of transition metal carbides, nitrides, or carbonitrides.^145–147^ This unique compositional flexibility, combined with their hierarchical structure, offers unprecedented opportunities for interface optimization in hydrogen storage applications. Initial breakthrough by Liu *et al.*^148^ demonstrated Ti_3_C_2_ MXene's remarkable capability in enhancing MgH_2_ systems, achieving 6.2 wt% hydrogen release at 300 °C and rapid absorption of 6.1 wt% H_2_ within 30 s at 150 °C through efficient electron transfer pathways enabled by the metallic conductivity of Ti_3_C_2_. Advancing this concept, Gao *et al.*^149^ developed few-layer Ti_3_C_2_T_*x*_ composites supporting highly dispersed nickel nanoparticles, where the synergistic coupling between MXene's conductive framework and catalytic Ni sites enabled hydrogen release of 5.83 wt% at 250 °C within 1800 s and absorption of 5 wt% hydrogen at 100 °C within 1700 s, maintaining stable performance through 10 cycles. Significant progress in MXene engineering was achieved by Peng *et al.*^150^ through Ni@C/FL-Ti_3_C_2_T_*x*_ nanosheets architecture, achieving 6.28 wt% H_2_ release within 5 minutes at 325 °C with remarkable cycling stability – 97.2% capacity retention after 50 cycles, attributed to the enhanced interfacial contact and confined nanostructure stability. A breakthrough in low-temperature performance came from Hu *et al.*,^151^ who constructed Ti_3_C_2_Cl_*x*_ MXene through molten salt etching, enabling room-temperature hydrogen absorption initiation while reducing release temperature by 200 °C through optimized surface termination groups. Further sophistication in MXene-based interfaces was demonstrated by Zhu *et al.*,^140^ who developed integrated MgH_2_–Ni@Ti-MX composites through chemical extraction of Al from Ti_3_AlC_2_ MAX phase (Fig. 10b1), achieving uniform particle distribution (200–300 nm) with continuous rings in SEAD patterns confirming MgO formation and scattered spots indicating Mg and Mg_2_Ni phases (Fig. 10b2 and b3), while high-angle annular dark-field imaging revealed well-dispersed bright spots (Fig. 10b4 and b5), leading to hydrogen release initiation at 305 °C and maximum desorption at 343 °C (Fig. 10b6). This progression in MXene-based interface engineering demonstrates their exceptional potential in magnesium-based hydrogen storage, combining inherent metallic conductivity with tunable surface chemistry and robust mechanical properties, though optimization of surface termination groups and long-term stability remain key challenges for practical implementation.

Beyond graphene and MXenes, alternative two-dimensional materials have demonstrated significant promise in interface engineering for magnesium-based hydrogen storage through their diverse compositional advantages. Early exploration by Zhang *et al.*^152^ established the potential of transition metal nanosheets through two-dimensional Fe layers interacting with magnesium hydride, where the engineered interfaces achieved activation energy reduction to 40.7 ± 1.0 kJ mol^−1^ and enabled hydrogen desorption initiation at 182.1 °C with 5.44 wt% release within 10 minutes at 300 °C. Building on this concept, Peng *et al.*^153^ engineered Ni nanoparticles supported on YC_*x*_F_*y*_ nanosheets, achieving further reduction in activation energy to 80.9 kJ mol^−1^ while maintaining exceptional cycling stability with 97.6% capacity retention through 50 cycles through the synergistic effects between the metal fluoride framework and catalytic sites. Significant advances in metal oxide-based interfaces were demonstrated by Li *et al.*,^141^ who developed TiB_2_ and NbB_2_ by using 2D TiNb_2_O_7_ nanosheets as an active precursor (Fig. 10c1) and obtained LMBH-5TNO (Fig. 10c2–c5) enabling remarkable 7.0 wt% capacity at 300 °C with minimal catalyst incorporation, attributed to the unique electronic structure and oxygen vacancy-mediated hydrogen diffusion pathways. And had a reversible capacity as high as 9.4 wt% remains even after 50 cycles, corresponding to a retention of 96% (Fig. 10c6). Ma *et al.*^154^ further advanced this direction through MnTiO_3_ nanosheets that effectively reduced the onset release temperature to 202 °C through optimized metal–oxygen coordination environments. A breakthrough in multi-component systems emerged through Lan *et al.*'s^155^ development of nickel and titanium dioxide co-modified carbon and nitrogen nanosheets, achieving exceptional low-temperature performance: 5.08 wt% absorption within 100 minutes at 40 °C and 6.17 wt% within 10 minutes at 125 °C, with reduced dehydrogenation activation energy of 83.1 kJ mol^−1^, attributed to the synergistic effects between stable TiO_2_ nanoparticles and reversible Ni^2+^/Ni^0^ interfacial processes. These diverse approaches in alternative two-dimensional materials complement graphene and MXene-based systems by offering unique opportunities for interface optimization through varied chemical compositions and coordination environments, though challenges remain in understanding and controlling the complex interfacial phenomena in these multi-component systems.

Two-dimensional materials demonstrate exceptional advantages in interface engineering compared to one-dimensional architectures through their distinctive structural features. Table 2 presents the hydrogen storage properties of two-dimensional materials, emphasizing the distinctions between few-layer structures and nanosheets in terms of rehydrogenation activation energy (*E*_ab_), dehydrogenation activation energy (*E*_de_), and cycling stability, and cycling stability. The extensive planar interfaces enable sophisticated control over catalytic reactions and charge transfer processes, while the layered geometry facilitates uniform distribution of active sites and enhanced particle-support interactions. However, this architectural design presents unique challenges in achieving comprehensive spatial confinement, as the two-dimensional configuration inherently limits the encapsulation of magnesium-based materials in the third dimension, affecting the overall efficiency of interface-mediated hydrogen storage processes.

**Table 2:** Hydrogen storage properties of two-dimensional materials

Materials	Structure	*E*_ab_ (kJ per mol H_2_)	*E*_de_ (kJ per mol H_2_)	Cycling stability	Ref.
GNS	Layer	78.4	129.2	—	139
B900-rGO-Mg	Layer	—	—	—	142
Ni-doped rGO-Mg	Layer	6–19	26–50	30	143
FeNi/rGO	Layer	42.3 ± 3.3	93.6 ± 7.3	50	144
Ti_3_C_2_ MXene	Layer	—	—	—	148
Ni_30_/FL-Ti_3_C_2_T_*x*_	Layer	—	96.36	10	149
Ni@C/FL-Ti_3_C_2_T_*x*_	Layer	—	74.7 ± 2.1	50	150
Ti_3_C_2_Cl_*x*_ MXene	Layer		141.07	—	151
Ni@Ti-MX	Layer	56 ± 4	73 ± 3.5	10	140
Ni_30_/YC_*x*_F_*y*_	Nanosheets	—	80.9 ± 3.7	50	153
LMBH-5TNO	Nanosheets	—	107 ± 1	50	141
MnTiO_3_	Nanosheets	—	72.06	50	154
(Ni, TiO_2_)CN	Nanosheets	41.9	83.1	100	155

### 5.3. Three-dimensional frameworks: space-optimized activity

Three-dimensional materials represent a fundamental advancement in interface architecture through their unique spatial organization and multidimensional pore designs. These frameworks simultaneously address multiple aspects of hydrogen storage performance by integrating the advantages of both one-dimensional channels and two-dimensional interfaces into interconnected networks. Their sophisticated architecture enables precise control over mass transport pathways and thermal management, while creating hierarchical interfaces that optimize both catalytic activity and structural stability during hydrogen cycling processes.

**Fig. 11:**
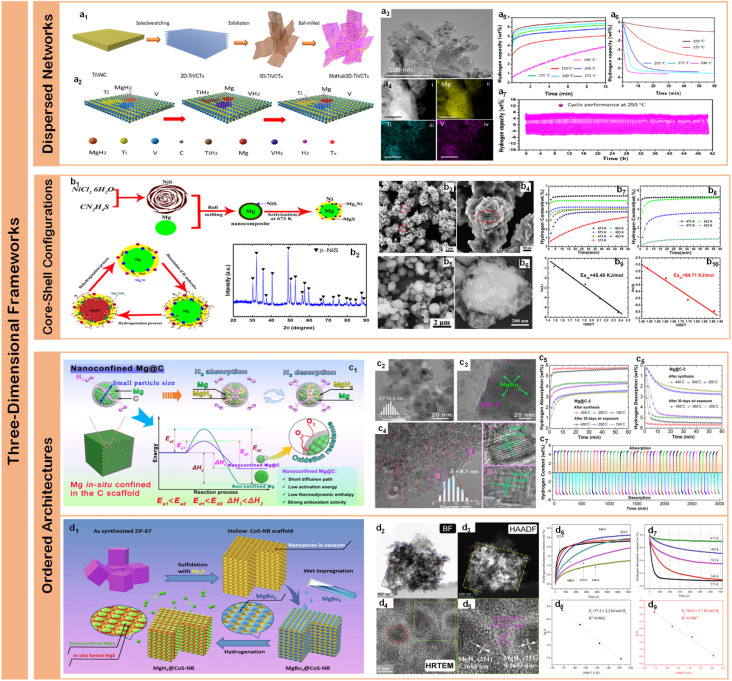
(a1) Schematic view of MXene preparation from the MAX phase by Al layers selecting etching, exfoliation of the 2D structure (2D-TiVCT_*x*_); (a2) and formation of the 3D delaminated (3D-TiVCT_*x*_) structure and composite with MgH_2_ nanoparticles; structural and morphology studies of MgH_2_@3D-TiVCT_*x*_: (a3) TEM micrographs, and (a4) HAADF and STEM-EDS mapping after cyclic performance. (a5 and a6) Hydrogenation and dehydrogenation kinetic performance at different temperatures, while (a7) sorption cyclic performance of MgH_2_@3D-TiVCT_*x*_ at 250 °C.^158^ (b1) Schematic diagram of the fabrication of the Mg–MgS–Mg_2_Ni–Ni nanocomposite and its first activization process at 673 K; (b2) XRD pattern of the NiS flowerlike particles; SEM (b3) and high magnification SEM (b4) images of the NiS flowerlike particles; (b5) SEM, (b6) high magnification SEM images of the nanocomposite; hydrogen absorption curves under 4 MPa hydrogen pressure (b7), desorption curves under 100 Pa (b8), plots of ln *k vs.* 1000/*T* for the hydrogenation (b9) and dehydrogenation (b10) of Mg–MgS–Mg_2_Ni–Ni nanocomposite.^159^ (c1) Schematic diagram of hydrogen absorption and desorption reaction mechanism of the Mg@C nanocomposites; (c2 and c3) TEM images of MgBu_2_ (inset: the particle size distribution histogram of MgBu_2_) and MgBu_2_@ZIF-8; HRTEM image (c4) (inset: the particle size distribution histogram of Mg); isothermal hydrogenation and dehydrogenation cycle curves of the Mg@C-3 (c5); isothermal hydrogenation curves (c6) and dehydrogenation curves (c7) of the Mg@C-3 after synthesis and after 30 days in air.^160^ (d1) Schematic illustrations of the nanoconfinement process for the synthesis of MgH_2_@CoS-NBs composite; typical bright field TEM image (d2); HAADF image (d3) and HRTEM micrographs (d4 and d5) for the MgH_2_@CoS-NBs composite, subjected to several H-cycles; isothermal hydriding (d6) and dehydriding (d7) profiles of Mg@CoS-NBs composite, and the corresponding ln *k vs.* 1000/*T* plots for determining the activation energy of hydrogenation (d8) and dehydrogenation (d9).^161^

Three-dimensional dispersed networks represent a distinctive approach in interface engineering through their random but interconnected spatial distribution of active components, fundamentally different from ordered frameworks or core–shell configurations. The key characteristic of these structures lies in their stochastic arrangement of catalytic sites and storage domains, creating a naturally formed network that enables multidirectional hydrogen diffusion paths. Zhou *et al.*^156^ demonstrated this concept through Fe–Ni modified 3D graphene, where the randomly distributed Fe–Ni catalytic sites across the graphene framework achieved remarkable kinetics – enabling hydrogen absorption of 6.35 wt% within 100 s and desorption of 5.13 wt% within 500 s at 300 °C, with stable capacity of 6.5 wt% maintained through multiple cycles. Similar transformative effects were observed in MXene-based dispersed systems. Zou *et al.*^157^ engineered an advanced 3D MgH_2_@Ti-MX architecture where the Ti-MX structure maintained its integrity without collapse after annealing. Their TEM analysis confirmed the preservation of structural features during cycling. The 60MgH_2_@Ti-MX composite demonstrated exceptional kinetics by absorbing 4.1 wt% H_2_ within 20 minutes under identical conditions, while maintaining remarkable cycling stability through 60 cycles at 200 °C. Building on this concept, Ali *et al.*^158^ developed sophisticated 3D Ti_3_C_2_T_*x*_ architectures to synthesize MgH_2_@3D-TiVCT_*x*_ composites (Fig. 11a1 and a2), achieving comprehensive enhancement: reduced dehydrogenation temperature (170 °C), rapid hydrogen absorption (6.5 wt% at 100 °C), and fast release (5.5 wt% at 300 °C) (Fig. 11a5 and a6). Most remarkably, this system demonstrated unprecedented cycling stability, maintaining performance through 180 cycles at 250 °C with minimal capacity decrease from 6.5 to 6.3 wt% (Fig. 11a7). TEM observations further confirmed the structural stability under high temperatures and pressures after extended cycling (Fig. 11a3 and a4). The success of these dispersed networks stems from their ability to maintain random but effective catalyst distribution throughout the storage matrix, providing abundant interfacial contacts while avoiding the structural complexity of ordered frameworks. However, this stochastic nature also presents challenges in precisely controlling local environments and ensuring uniform performance across the material.

Core–shell configurations manipulate hydrogen storage behavior through rationally designed dual-functional interfaces, where each component serves a distinct yet complementary role in the storage process – fundamentally different from randomly dispersed catalysts or ordered porous frameworks. Zhang *et al.*^162^ demonstrated this interfacial synergy through an innovative core–shell structure combining Ni_3_N with N-doped carbon (Ni_3_N@NC). The architecture featured precisely controlled dimensions – a 16 nm Ni_3_N core encapsulated by a 2 nm nitrogen-doped carbon shell, enabling significantly reduced initial desorption temperature (175 °C) and rapid hydrogen release (6.0 wt% H_2_ within 20 minutes at 325 °C). Kinetic analysis revealed consistent Avrami exponents (*n* = 1.87–2.10) across different temperatures, indicating stable phase transformation mechanisms maintained by the core–shell interface. Advancing this concept through hollow structure design, Lai and Aguey-Zinsou^163^ developed Cu_2_S hollow spheres for confining Mg(BH_4_)_2_, with precisely controlled dimensions (100–800 nm diameter, 35 nm wall thickness). The engineered shell structure created efficient hydrogen diffusion pathways that enabled complete absorption within 150 minutes, while the enhanced interfacial interactions facilitated hydrogen desorption at 219 °C – a significant reduction from the 250 °C threshold observed in conventional physical mixtures. Noble metal integration revealed additional interfacial effects, as demonstrated by Lu *et al.*^164^ through Mg@Pt composites with uniformly distributed 3 nm Pt nanoparticles, achieving 6 wt% absorption within 2 hours at 225 °C and complete 6.5 wt% desorption within 20 minutes at 350 °C through platinum's unique “spillover” mechanism. Panda *et al.*^165^ extended this approach with Mg@Pd architectures, optimizing interfacial contact through precise structural control verified by electron tomography. Xie *et al.*^159^ developed an innovative flower-like NiS templated system (Fig. 11b1 and b2), where magnesium hydride was encapsulated by hierarchically structured NiS shells through a precisely controlled self-assembly process (Fig. 11b3–b6). This rationally designed core–shell architecture, with NiS nanosheets forming a protective and catalytic outer layer around the magnesium core, exhibited rapid kinetics (3.5 wt% H_2_ absorption within 10 minutes at 150 °C, 3.1 wt% H_2_ desorption within 10 minutes at 300 °C) (Fig. 11b7–b10) with significantly reduced activation energies (45.45 and 64.71 kJ mol^−1^ for absorption and desorption). The success of these core–shell configurations stems from their unique interfacial design – the shell component provides selective hydrogen transport channels while protecting against environmental degradation, and the core maintains intimate contact with catalytic sites while preserving high storage capacity, offering controlled reaction pathways distinct from both dispersed and ordered framework systems.

Ordered three-dimensional frameworks represent an advanced architectural strategy that transcends core–shell configurations, offering unprecedented control over hydrogen storage performance through systematically engineered pore networks and precisely organized diffusion channels. These sophisticated architectures integrate the advantages of both spatial confinement and catalytic functionality into hierarchically ordered structures. Shao *et al.*^166^ pioneered this approach through integrating magnesium hydride within three-dimensionally ordered microporous titanium dioxide (3DOM TiO_2_) frameworks, where the precisely designed pore architecture (∼150 nm) created hierarchical diffusion pathways. The resulting composite, featuring magnesium hydride nanoparticles uniformly distributed throughout the ordered TiO_2_ framework, demonstrated remarkable performance: achieving rapid hydrogen absorption (4.17 wt% H_2_ within 1800 s at 100 °C) and desorption (5.75 wt% H_2_ within 1000 s at 300 °C). Chen *et al.*^167^ extended this concept to 3DOM Nb_2_O_5_, where nanoparticles (5–15 nm) wrapped in amorphous carbon layers demonstrated exceptional kinetics, absorbing 6.35 wt% H_2_ within 500 s at 175 °C and desorbing 6.95 wt% H_2_ within 300 s at 300 °C. Metal–organic frameworks (MOF) have emerged as particularly promising templates for creating sophisticated ordered architectures. Elsabawy *et al.*^168^ developed a nano-architectonic encapsulation approach combining reduced graphene oxide (rGO) nanosheets with hollow carbon nano-spheres (HCNS), achieving 6.45 wt% hydrogen uptake at 350 °C under 14 bar H_2_. Xie *et al.*^169^ utilized 3D porous flower-like Ni/Zn-MOFs to prepare Ni_3_ZnC_0.7_/Ni/ZnO composites, achieving reduced dehydrogenation activation energy (93.06 kJ mol^−1^) with retention rates exceeding 98% after 20 cycles. Xing *et al.*^160^ developed an innovative approach encapsulating Bu_2_Mg nanosheets in ZIF-8 to form Mg@C nanocomposites (Fig. 11c1) with precisely controlled particle sizes (8.7 nm average) (Fig. 11c2–c4), achieving high loading capacity (76.3%) while maintaining exceptional stability. Their Mg@C-3 composite demonstrated remarkable performance at moderate temperatures with outstanding capacity retention (97.1% absorption, 96.7% desorption) through 50 cycles attributed to effective nano-confinement and enhanced interfacial reactions (Fig. 11c5–c7). A significant advancement in ordered three-dimensional architectures was achieved by Ma *et al.*,^161^ who developed a template-consumption strategy for precise confinement of MgH_2_ within CoS-NBs frameworks (Fig. 11d1). Their systematic approach resulted in highly uniform distribution of 5–10 nm MgH_2_ crystallites throughout the ordered box-like scaffold structure (Fig. 11d2–d5), demonstrating superior control over both spatial organization and interface engineering. This architectural design enabled unprecedented thermodynamic tuning, with hydrogenation and dehydrogenation enthalpies optimized to −65.6 ± 1.1 and 68.1 ± 1.4 kJ per mol H_2_ respectively. Moreover, the synergistic effects between nanoscale confinement and catalytic interfaces led to substantially reduced activation barriers of 57.4 ± 2.2 and 120.8 ± 3.2 kJ per mol H_2_ for absorption and desorption processes (Fig. 11d6–d9). These ordered architectures demonstrate unique advantages through their ability to precisely control spatial organization at multiple length scales, creating optimized pathways for hydrogen transport while maintaining structural stability through extended cycling.

Table 3 summarizes the hydrogen storage properties of three-dimensional materials, highlighting the impact of different structural configurations on hydrogen storage performance. Dispersed structures typically exhibit higher cycling stability, with some materials reaching up to 180 cycles, but their hydrogen absorption and desorption kinetics still require further optimization. The core–shell structures offer moderate hydrogen storage performance while ordered structures demonstrate excellent hydrogen absorption and desorption kinetics, with some materials achieving absorption activation energies as low as 22.8–23.6 kJ mol^−1^ and desorption activation energies around 72.8–81.73 kJ mol^−1^. Additionally, they demonstrate relatively good cycling stability, reaching up to 50 cycles. These findings underscore the critical role of structural design in optimizing hydrogen storage performance. The systematic investigation of three-dimensional frameworks – from dispersed networks to core–shell configurations and ordered architectures – has established fundamental principles for interface engineering in magnesium-based hydrogen storage systems. Each architectural strategy demonstrates distinct advantages: dispersed structures enable stochastic but effective distribution of active sites while maintaining structural flexibility; core–shell configurations achieve precise control over interfacial processes through dual-functional design; and ordered architectures provide optimized pathways for mass transport through hierarchical channel organization. The synergistic integration of structural design and compositional modulation at the nanoscale has enabled unprecedented performance improvements: reduction of operating temperatures below 150 °C, achievement of practical absorption/desorption rates (>6 wt% within minutes), and exceptional cycling stability through hundreds of cycles. These advances stem from the comprehensive understanding and control of interfacial phenomena – from electron transfer and hydrogen spillover at metal–metal interfaces to selective mass transport through engineered channels. However, significant challenges remain in achieving practical implementation: the trade-off between high magnesium loading (>80 wt%) and interface stability, the complexity of scalable synthesis for sophisticated architectures, and the need for mechanistic understanding of interface evolution during extended cycling.

**Table 3:** Hydrogen storage properties of three-dimensional materials

Materials	Structure	*E*_ab_ (kJ per mol H_2_)	*E*_de_ (kJ per mol H_2_)	Cycling stability	Ref.
Fe–Ni/3d graphene	Dispersed	—	—	7	156
Ti-MX	Dispersed	—	—	60	157
3D-TiVCT_*x*_	Dispersed	—	—	180	158
Ni_3_N@NC	Core–shell	—	100	—	162
Mg@Pt	Core–shell	82.4	152.8	—	164
Mg@Pd	Core–shell	—	—	—	165
NiS	Core–shell	45.45	64.71	20	159
3DOM TiO_2_	Ordered	22.8	72.8	10	166
3DOM Nb_2_O_5_	Ordered	23.61	81.73	10	167
rGO-HCNS	Ordered	—	—	—	168
Ni_3_ZnC_0.7_/Ni/ZnO	Ordered	44.85	93.06	20	169
CoS-NBs	Ordered	65.6 ± 1.1	68.1 ± 1.4	10	161
Mg@C	Ordered	53.1	74.2	50	160

## 6. Conclusions and perspectives

Interface engineering has emerged as a paradigm-shifting strategy that fundamentally transforms the landscape of magnesium-based hydrogen storage through unprecedented control over atomic-scale interactions and energy landscapes. This review establishes a sophisticated framework demonstrating how strategic interface design orchestrates multiple scales of functionality: from atomic-level electronic modulation that reduces hydrogen dissociation barriers, through nanoscale architectural control that creates efficient diffusion pathways, to mesoscale spatial organization that optimizes both thermodynamic and kinetic performance. The field has witnessed remarkable breakthroughs through interface-mediated mechanisms: quantum confinement effects at metal–metal interfaces destabilize Mg–H bonds by precisely tuning electron density distributions, engineered support interfaces maintain structural integrity while facilitating rapid hydrogen transport through rationally designed channels, and hierarchical catalytic interfaces create synergistic effects that simultaneously enhance multiple performance metrics. Our comprehensive analysis reveals that interface engineering has enabled unprecedented achievements – reduction of dehydrogenation temperatures by over 150 °C (from >300 °C to <150 °C), dramatic lowering of activation energies by nearly 100 kJ mol^−1^ (from ∼160 kJ mol^−1^ to ∼60–80 kJ mol^−1^), and realization of practical hydrogen storage rates exceeding 6 wt% within minutes while maintaining exceptional cycling stability through hundreds of cycles. Most significantly, the strategic integration of structural architecture and compositional design has unlocked new mechanistic pathways: the formation of “hydrogen highways” through precisely engineered interfaces enables directional hydrogen diffusion, the creation of electronic “spillover bridges” facilitates hydrogen dissociation at dramatically reduced energy barriers, and the development of “smart interfaces” that can dynamically respond to hydrogen cycling provides unprecedented control over long-term stability. These fundamental advances in interface science not only establish new design principles for magnesium-based hydrogen storage but also offer broader insights into controlling gas–solid interactions through interface engineering.

Despite these remarkable achievements, several critical challenges remain at the frontier of interface engineering for magnesium-based hydrogen storage systems. The fundamental trade-off between interface stability and catalytic activity presents a primary challenge: while increased interface density enhances reaction kinetics, it simultaneously introduces potential degradation pathways through interface evolution during hydrogen cycling. Our analysis reveals that current nanoconfinement strategies struggle to maintain high magnesium loading (>80 wt%) while preserving interface stability, particularly when operating temperatures exceed 150 °C for practical absorption/desorption rates. The development of scalable synthesis methods for sophisticated interface architectures faces inherent challenges in controlling uniformity and reproducibility across multiple length scales. Most critically, the dynamic evolution of interfaces during long-term cycling – including catalyst migration, phase transformation, and structural reorganization – requires advanced *in situ* characterization techniques for mechanistic understanding. These challenges point toward several promising research directions: First, the development of “adaptive interfaces” that can accommodate structural evolution while maintaining catalytic functionality through precise control of phase transformation pathways. Second, the exploration of novel hybrid architectures combining multiple functional interfaces – such as integrating catalytic metals with heteroatom-doped frameworks – to create synergistic effects beyond simple addition of individual components. Third, the advancement of *in situ*/*operando* characterization techniques capable of probing interface dynamics during hydrogen cycling to establish structure–property relationships and guide rational design. Finally, the development of computational methods that can predict interface stability and evolution under realistic operating conditions, enabling accelerated materials discovery and optimization.

The convergence of emerging technologies and interface engineering opens unprecedented opportunities for advancing magnesium-based hydrogen storage systems. Advanced synthesis strategies, particularly those leveraging precise molecular assembly and controlled phase transformation, offer promising routes for creating sophisticated interface architectures. The development of “programmable interfaces” through molecular-level control of surface chemistry and electronic structure could enable dynamic optimization of hydrogen storage performance under varying operating conditions. Integration of artificial intelligence and high-throughput experimentation presents transformative potential for accelerating interface design and optimization: machine learning algorithms can predict optimal interface compositions and architectures, while automated synthesis platforms enable rapid validation and iteration. At the materials level, several emerging directions show particular promise: First, the development of hierarchical interface architectures that simultaneously optimize multiple performance metrics through spatial organization across different length scales. Second, the creation of “smart catalytic interfaces” that can reversibly switch between different functional states to regulate hydrogen storage kinetics. Third, the engineering of interface stability through controlled formation of beneficial secondary phases during cycling. Most significantly, recent advances in atomic-scale characterization techniques, particularly *in situ* electron microscopy and spectroscopy methods, enable unprecedented insights into interface evolution during hydrogen cycling. This mechanistic understanding, combined with theoretical modeling across multiple time and length scales, provides a powerful framework for rational interface design. From an applications perspective, the integration of interface-engineered materials into practical hydrogen storage systems requires careful consideration of system-level requirements: thermal management during hydrogen cycling, mechanical stability under repeated volume changes, and cost-effective scalability of materials synthesis. The development of prototype systems incorporating these advanced materials will be crucial for demonstrating practical viability and guiding further optimization.

## Author contributions

Han Jiang contributed to the original draft preparation and participated in the review and editing of the manuscript. Zhao Ding was responsible for the conceptualization, supervision, funding acquisition, formal analysis, and manuscript review and editing. Yuting Li, Guo Lin, Shaoyuan Li, and Wenjia Du contributed to the review and editing of the manuscript. Yu'an Chen participated in the formal analysis, supervision, and manuscript review and editing. Leon L. Shaw contributed to the conceptualization and formal analysis. Fusheng Pan provided the conceptualization of the work.

## Conflicts of interest

The authors declare that there is no conflict of interests regarding the publication of this article.

## Data Availability

No primary research results, software or code have been included and no new data were generated or analysed as part of this review.
